# Phytochemical Profiling, In Vitro α-Amylase Inhibitory and Antioxidant Activities of *Madhuca longifolia* Leaf and Bark Extracts with Molecular Docking Analysis

**DOI:** 10.3390/plants15193059

**Published:** 2026-10-07

**Authors:** Parvej Ahmad, Mohd. Waiz, Tabrez Faruqui, Supriya Srivastava, Khurshid Ahmad, Abbas Ali Mahdi, M. Salman Khan

**Affiliations:** 1 Stem Cell Research Center (SCRC), Department of Hematology, Sanjay Gandhi Postgraduate Institute of Medical Science (SGPGIMS), Lucknow 226014, Uttar Pradesh, India; contactpahmad@gmail.com; 2 Department of Bioscience, Integral University, Lucknow 226026, Uttar Pradesh, India; mohdwaiz032@gmail.com; 3 Department of Personalized and Molecular Medicine, Era University, Lucknow 226002, Uttar Pradesh, India; tabrezfaruqui@gmail.com; 4 Department of Health Informatics, College of Applied Medical Sciences, Qassim University, Buraydah 51452, Saudi Arabia; s.srivastava@qu.edu.sa; 5 Department of Biochemistry, Era University, Lucknow 226002, Uttar Pradesh, India; 6 Department of Biotechnology, Era University, Lucknow 226002, Uttar Pradesh, India

**Keywords:** *Madhuca longifolia*, antioxidant activity, α-amylase inhibition, phytochemical profiling, GC-MS, molecular docking

## Abstract

Plant-derived phytochemicals are increasingly investigated for their antioxidant and enzyme-inhibitory activities as potential sources of bioactive compounds. The present study evaluated the phytochemical composition, antioxidant activity, and in vitro α-amylase inhibitory activity of *Madhuca longifolia* leaf and bark extracts, followed by GC-MS profiling and exploratory computational analyses of the methanolic leaf fraction. Moreover, we elucidated the ADME properties of GC-MS-putatively identified compounds and their computational molecular interactions with α-amylase. The leaves and barks were sequentially extracted using n-hexane, DCM, EtOAc, MeOH, and water. The qualitative phytochemical analysis and antioxidant activity were determined via DPPH, ABTS, and FRAP assays. Furthermore, the MeOH fraction of *M. longifolia* leaves was subjected to GC-MS analysis to identify the secondary metabolites. The putatively identified compounds were further subjected to ADME analysis and computational molecular interaction studies with α-amylase. Phytochemical screening indicated the presence of several tested phytochemical classes in the MeOH and aqueous fractions of the leaf and bark extracts. The MeOH and aqueous fractions of the leaf extract showed IC_50_ values of 8.67 and 10.51 µg/mL, respectively, against DPPH, whereas the corresponding bark fractions showed IC_50_ values of 8.21 and 8.15 µg/mL, respectively. A similar trend was observed in the ABTS assay, where the MeOH and aqueous fractions of the leaf extract exhibited IC_50_ values of 4.32 and 4.68 µg/mL, respectively, while the corresponding bark fractions showed IC_50_ values of 4.44 and 5.40 µg/mL, respectively. The maximum α-amylase inhibitory potential was observed in the MeOH fraction of the leaf extract, and global nonlinear enzyme kinetic analysis showed a pattern consistent with competitive inhibition. The in silico molecular docking analysis predicted that 6-methylheptanoic acid from the MeOH fraction of the leaf extract may interact with the catalytic site of human α-amylase, with a Glide score of −6.132. In conclusion, the findings provide preliminary evidence of antioxidant and in vitro α-amylase inhibitory activities of the MeOH fraction of *M. longifolia* leaf extract and support its consideration as a preliminary lead for further investigation. Further studies involving quantitative chemical characterization, isolation and validation of individual constituents, cellular and pharmacodynamic evaluation, experimental toxicity assessment, and in vivo efficacy studies are required to establish their relevance to glucose metabolism and diabetes-related outcomes.

## 1. Introduction

In the 21st century, diabetes mellitus (DM) has become a major worldwide public health challenge, characterized by dysregulated carbohydrate metabolism and subsequent high fasting blood sugar concentrations due to impaired insulin secretion and action. DM is associated with severe complications, including retinopathy, neuropathy, nephropathy, and cardiovascular diseases [1,2,3]. The global burden of diabetes is increasing at an alarming rate. According to the International Diabetes Federation (IDF) report, approximately 11.1% of the adult world population (590 million) aged 20–79 years will live with DM in 2025, and this number is projected to rise to 853 million by 2050, representing a 46% increase compared to the current situation, meaning nearly 1 in 8 adults will be affected globally [4]. In these cases, type 2 diabetes (T2DM) accounts for more than 90% of cases, largely driven by urbanization, inactive lifestyles, unhealthy dietary patterns, and growing rates of obesity [5,6]. India is experiencing one of the world’s largest diabetes epidemics, often referred to as the “diabetes capital of the world.” In 2024, about 89.8 million (10.5% prevalence) Indian adults aged 20–79 years were living with diabetes, making India the country with the second-highest number of cases globally, after China. Equally concerning is the fact that 43% of these individuals–nearly 38.6 million–remain undiagnosed, highlighting substantial gaps in screening and healthcare access [4]. In India, the number of adults with diabetes is projected to reach approximately 156.7 million by 2050, compared with about 89.8 million in 2024 [4]. T2DM progression is driven by multiple interrelated risk factors, including impaired insulin secretion resulting from β-cell dysfunction, physical inactivity, obesity, insulin resistance, hypertension, genetic predisposition, aging, ethnicity, family history of diabetes, and environmental factors, as well as dysregulation of carbohydrate-metabolizing enzymes, such as α-amylase and α-glucosidase [7].

Several studies have reported that altered carbohydrate metabolizing enzymes, including α-amylase and α-glucosidase, impair the digestion of dietary carbohydrates and enhance postprandial glucose levels. Persistent hyperglycemia promotes the non-enzymatic glycation of proteins, lipids, and nucleic acids, leading to the formation of advanced glycation end products (AGEs). The accumulation of AGEs contributes to the pathogenesis of T2DM and associated complications by exacerbating oxidative stress and inflammation [6,8]. Thus, pharmacological inhibition of α-amylase and α-glucosidase has been identified as an effective therapeutic approach to manage hyperglycemia [9,10].

Antidiabetic medications from synthetic sources that target α-amylase and α-glucosidase can prevent hyperglycemia. Despite their efficacy, these drugs fail to reverse complications and can cause adverse effects [11]. Growing evidence from around the world has established that plants and their secondary metabolites possess considerable therapeutic potential against various diseases, including T2DM and its associated complications [12]. Therefore, natural sources are being investigated for bioactive compounds with anti-diabetic potential. Therapeutic approaches targeting oxidative stress, reducing glucose absorption, and carbohydrate metabolizing enzymes hold promise for effective management of hyperglycemia [10,13].

**Figure 1:**
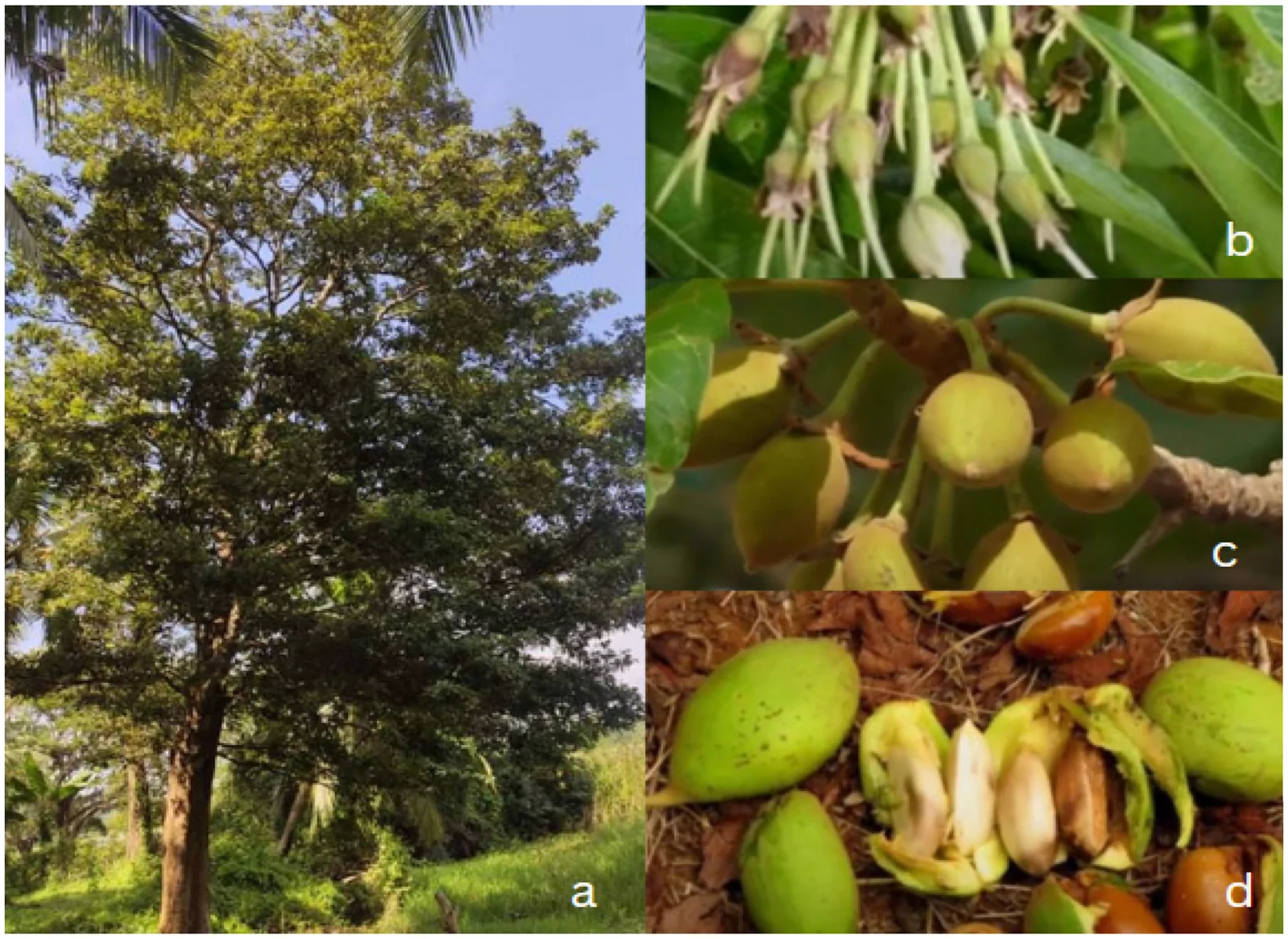
Illustrative representations of *M. longifolia* (Mahua): (**a**) tree, leaf, (**b**) flower, (**c**) fruits, and (**d**) seeds. Adapted from Hippola et al. (2023), licensed under CC BY 4.0 [23].

The butternut tree, or *Madhuca longifolia* (*M. longifolia*), belongs to the Sapotaceae family and is extensively distributed in Nepal, India, and Sri Lanka. The common Indian names for *M. longifolia* are Mahua, Mahwa, or Illuppai, which are majorly found in warm, moist climates. *M. longifolia* is a deciduous tree with a maximum height of 20 m [14] (Figure 1). Its bark, leaves, flowers, and seeds, phytochemical substances, including volatile oil, flavonoids, tannins, histidine, glutamic acid, vitamins A, C, β-carotene, and xanthophylls, are excessively used for medical applications [15,16]. *M. longifolia* extracts showed hepatoprotective and nephroprotective activity in acetaminophen-induced damage of renal and hepatic tissue [17]. It also possesses antioxidant, anti-inflammatory, antimicrobial, neuroprotective, wound healing, and skin care activities [18,19]. Previously, the presence of phytoconstituents, such as flavonoids, saponins, and polyphenols, in *M. longifolia* has been reported [17,20]. Previous studies have reported the biological activities of *M. longifolia* extracts, including α-amylase inhibitory activity, and individual constituents of the plant have also been investigated in relation to antidiabetic effects. Therefore, α-amylase inhibition by *M. longifolia* should not be considered novel in itself. The present study instead focuses on a comparative evaluation of sequentially prepared leaf and bark extracts, integrating qualitative phytochemical screening, antioxidant assays, and in vitro α-amylase inhibition with GC-MS profiling of the methanolic leaf fraction and exploratory computational analyses. This integrated approach provides a comparative characterization of the tested extracts and generates hypotheses regarding phytochemical constituents that may contribute to the observed in vitro activity [21,22]. In light of these reported properties, we comparatively evaluated sequentially prepared *M. longifolia* leaf and bark extracts for antioxidant and in vitro α-amylase inhibitory activities. The methanolic leaf fraction was further subjected to GC-MS profiling and exploratory computational analysis to investigate potential interactions of its reported constituents with α-amylase. Thus, the principal contribution of the present study is the comparative and integrated characterization of sequential leaf and bark extracts of *M. longifolia*, combining extract-level antioxidant and in vitro α-amylase inhibitory assessment with chemical profiling and exploratory molecular analysis.

## 2. Results and Discussion

### 2.1. Phytochemical Screening and Percentage Extract Yield

Natural products and their derivatives are traditionally recognized as therapeutic agents and structural heterogeneity. The process of drug discovery requires the identification of new chemical entities possessing druggability and medicinal chemistry attributes. Prior to high-throughput screening, 80% of pharmaceutical compounds were derived from natural products and their derivatives, including semi-synthetic analogs [24]. Therefore, we performed Soxhlet-based sequential extraction of *M. longifolia* leaf and bark using n-hexane, DCM, EtOAc, MeOH, and water. *M. longifolia* leaf and bark yielded sequential solvent fractions, with the corresponding extraction yields presented in Table 1. We calculated the % yield of all the extracts of *M. longifolia* leaf, the maximum yield % was observed in the aqueous (12.79%) and methanolic (9.84%) fractions (Table 1). On the other hand, in bark, the highest yield percentage was found in MeOH (5.26%) and aqueous (4.52%) fractions.

Qualitative phytochemical screening provides preliminary information on the classes of secondary metabolites present in plant extracts. The phytochemical composition of plants may vary with environmental conditions, including temperature, salt, radiation, water availability, developmental stage, seasonal fluctuations, and growth location [25]. Therefore, we performed qualitative phytochemical screening of all fractions obtained from *M. longifolia* leaves and bark. Qualitative phytochemical screening indicated the presence of several phytochemical classes across different fractions. Alkaloids, flavonoids, glycosides, phenols, and terpenoids were detected in the leaf extract, whereas the bark fractions showed the presence of tannins, saponins, alkaloids, flavonoids, glycosides, phenols, terpenoids, cardiac glycosides, and steroids in selected fractions (Table 2). Previous studies have reported that MeOH and aqueous fractions can contain a broader range of extractable secondary metabolites than nonpolar and semipolar solvent fractions [8,26]. Therefore, our results are consistent with those of prior reports.

### 2.2. DPPH Radical Scavenging Potential of M. longifolia Leaf and Bark Extract

**Figure 2:**
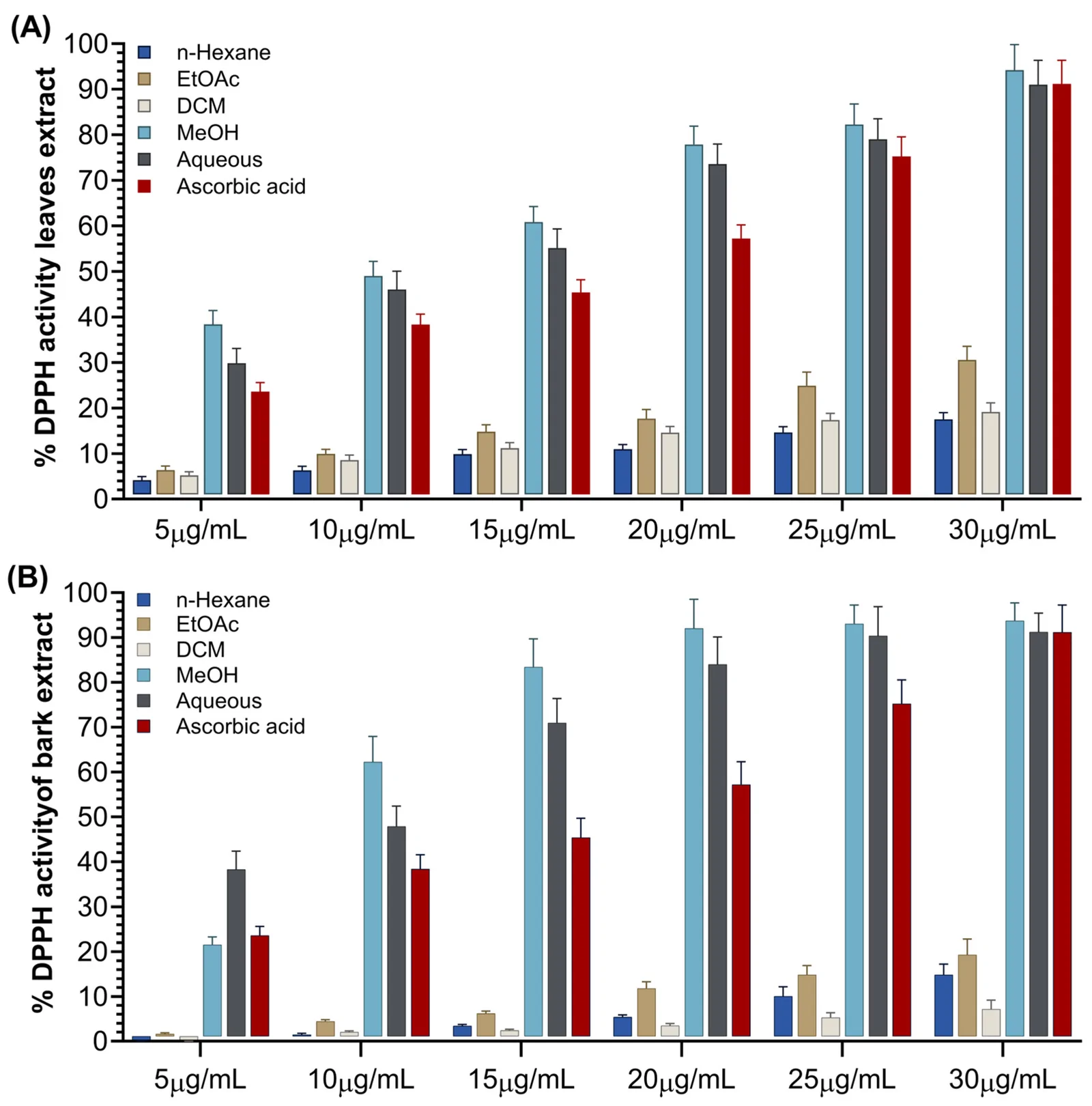
In vitro DPPH radical-scavenging potential of *M. longifolia* (**A**) leaf and (**B**) bark extracts of n-hexane, DCM, EtOAc, MeOH, and water. The bar graph represents the % DPPH quenching ability of each extract along with the standard ascorbic acid. The values are mean ± SEM of technical triplicate (*n* = 3) assays from a single extraction batch.

Altered carbohydrate metabolism contributes to persistent hyperglycemia, which promotes oxidative stress and disrupts insulin signaling pathways by inhibiting the phosphorylation of insulin receptor substrate-1. This suppresses the activation of phosphoinositide 3-kinase PI3 and protein kinase B, thereby impairing glucose transporter-4 translocation to the plasma membrane and reducing cellular glucose uptake [27,28]. Thus, increased oxidative stress and elevated postprandial glucose levels contribute to the pathogenesis of diabetes [29]. Natural antioxidants have attracted considerable interest because they can neutralize reactive oxygen species (ROS), thereby reducing oxidative stress associated with diabetes and helping to control postprandial hyperglycemia [30,31]. Therefore, we investigated the antioxidant potential of distinct fractions of *M. longifolia* leaf and bark extracts by inhibiting DPPH and ABTS radicals and ferric reducing efficacy. The percent inhibition of free radicals by each fraction is shown in Figure 2A. The aqueous and MeOH fractions of the leaf extract exhibited DPPH radical-scavenging activity, with IC_50_ values of 10.51 µg/mL (95% CI: 9.166–11.85 µg/mL) and 8.67 µg/mL (95% CI: 7.178–10.10 µg/mL), respectively (Figure 2A; Table 3). Ascorbic acid exhibited 91.17% inhibition, with an IC_50_ value of 13.70 µg/mL (95% CI: 11.76–15.64 µg/mL). Both the MeOH and aqueous fractions of the leaf extract had lower IC_50_ values than the standard. Similarly, the MeOH and aqueous fractions of the bark extract showed DPPH radical-scavenging activity, with IC_50_ values of 8.21 µg/mL (95% CI: 7.660–8.788 µg/mL) and 8.15 µg/mL (95% CI: 6.822–9.447 µg/mL), respectively (Figure 2B; Table 3) [32]. Both the MeOH and aqueous fractions of the leaf extract had lower IC_50_ values than the standard (ascorbic acid). Our findings suggest that these fractions contain specific secondary compounds that may contribute to free-radical neutralization and may contribute to the reduction in oxidative stress. In contrast, n-hexane, DCM, and EtOAc exhibited the lowest DPPH radical-scavenging activity, with maximum inhibition values of 14.81%, 19.30%, and 7.17%, respectively. The pronounced DPPH radical-scavenging activity observed in the MeOH and aqueous fractions of both leaf and bark extracts may be associated with the presence of phytochemical classes such as phenols, alkaloids, glycosides, flavonoids, and terpenoids, as indicated by the preliminary phytochemical screening and supported by previous reports [33].

### 2.3. ABTS Radical Scavenging Potential of M. longifolia Leaf and Bark Extract

**Figure 3:**
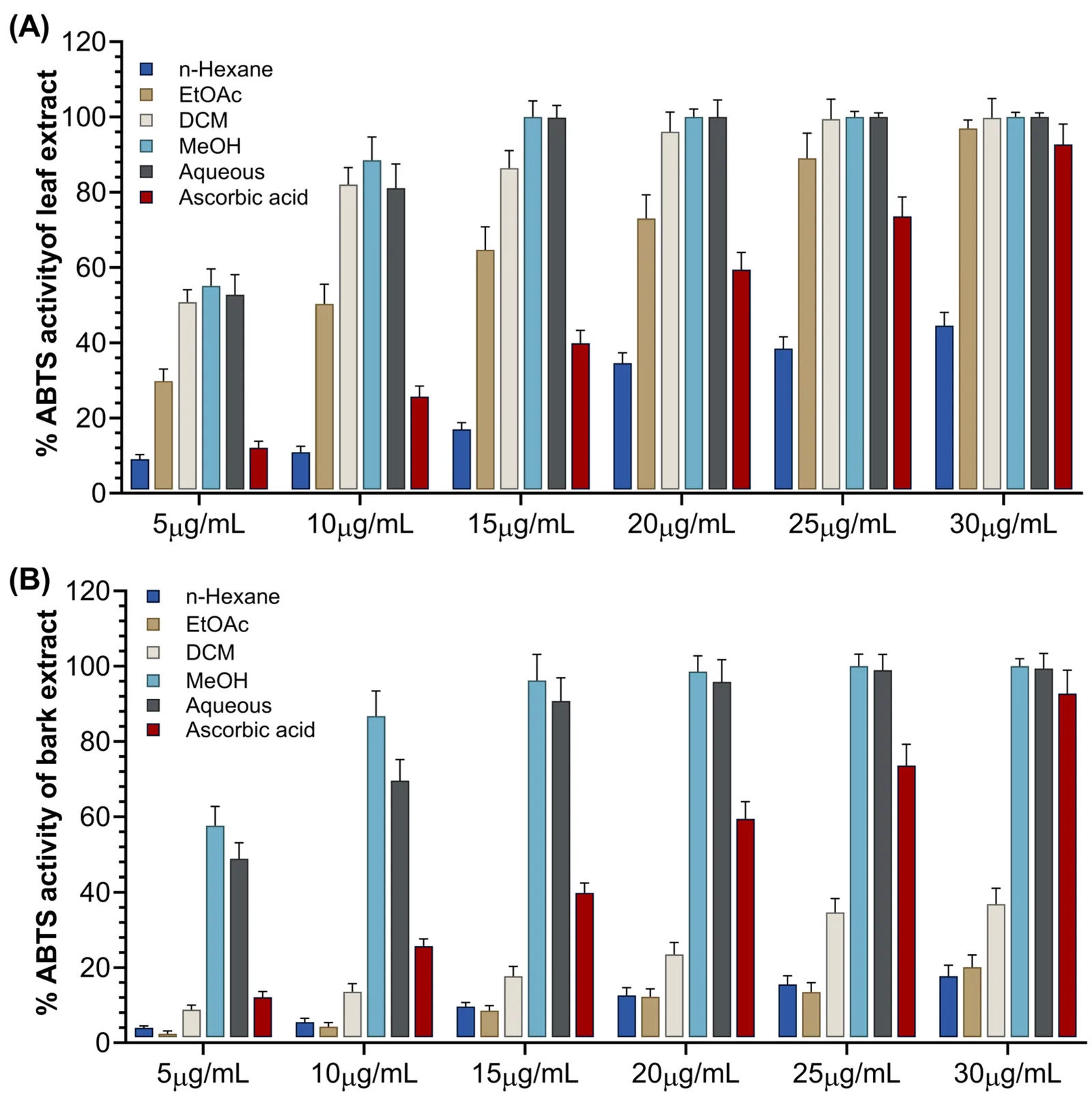
In vitro ABTS radical quenching ability of *M. longifolia* (**A**) leaf and (**B**) bark extract of n-hexane, DCM, EtOAc, MeOH, water, and ascorbic acid. The bar graph represents the percentage ABTS radical-scavenging activity of each extract along with the standard ascorbic acid. The values are mean ± SEM of technical triplicate (*n* = 3) assays from a single extraction batch.

We found that all fractions of leaf extract, except n-hexane, showed ABTS radical-scavenging activity in a dose-dependent manner. The MeOH and aqueous fractions exhibited the highest ABTS radical-scavenging potential, with IC_50_ values of 4.32 µg/mL (95% CI: 4.005–4.641 µg/mL) and 4.68 µg/mL (95% CI: 4.299–5.081 µg/mL), respectively. The DCM and EtOAc fractions also exhibited marked inhibition of ABTS radicals, with IC_50_ values of 4.82 µg/mL (95% CI: 4.445–5.219 µg/mL) and 9.38 µg/mL (95% CI: 8.319–10.47 µg/mL), respectively (Figure 3A; Table 3). Moreover, the positive control, ascorbic acid, exhibited an IC_50_ value of 10.81 µg/mL (95% CI: 9.457–12.22 µg/mL). In contrast, the MeOH and aqueous fractions of *M. longifolia* bark extract also exhibited ABTS radical-scavenging activity in a dose-dependent manner, with IC_50_ values of 4.44 µg/mL (95% CI: 3.930–4.870 µg/mL) and 5.40 µg/mL (95% CI: 4.620–6.136 µg/mL), respectively (Figure 3B; Table 3). The n-hexane, DCM, and EtOAc fractions were less effective in scavenging ABTS radicals. Both MeOH and aqueous fractions of *M. longifolia* bark extract showed lower IC_50_ values than the standard. These results are consistent with those of a previous study [18]. Our findings suggest that the MeOH and aqueous fractions of *M. longifolia* leaves and bark possess considerable free radical-scavenging potential compared to that of the standard. Free radicals have unpaired electrons by which they interact and modify lipids, proteins, and nucleic acids. Ultimately, they induce oxidative stress, which contributes to several diseases, including diabetes. Therefore, taking external antioxidants in the form of diet or medications can aid in oxidative stress management. Previous studies from our group and others have shown that plants contain several bioactive compounds such as phenols and flavonoids, which can serve as vital antioxidants because of their ability to neutralize free radicals by donating electrons [8,34].

### 2.4. Leaf and Bark Extract of M. longifolia Showed Strong Ferric Reducing Potential

**Figure 4:**
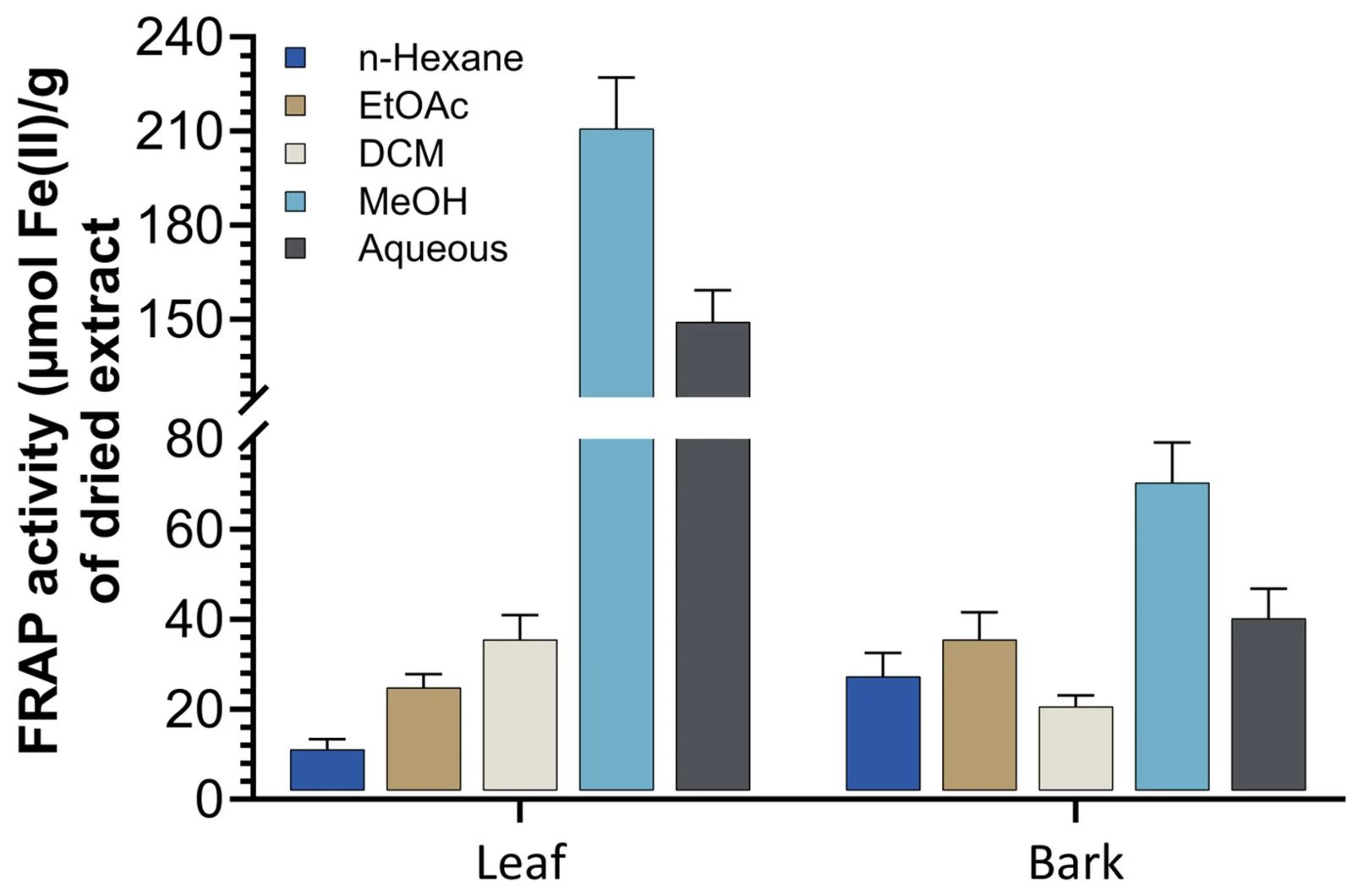
Ferric reducing antioxidant power (FRAP) of *M. longifolia* leaf and bark extracts. The values are mean ± SEM of technical triplicate (*n* = 3) assays from a single extraction batch.

The total antioxidant potential of the extracts was evaluated using the ferric reducing antioxidant power (FRAP) assay. Here, the antioxidant compounds act as reducing agents, donating electrons to reduce Fe^3+^-TPTZ into Fe^2+^-TPTZ. Our results demonstrated that the FRAP values were higher in the MeOH (210.78 ± 8.03µmol Fe(II) equivalents/g dry extract) and aqueous (149.07 ± 6.51µmol Fe(II) equivalents/g dry extract) fractions of *M. longifolia* leaf extract. However, the rest of the fractions reported low FRAP values, such as 11.09 ± 2.34, 35.45 ± 4.02, and 24.84 ± 3.22 µmol Fe(II) equivalents/g dry extract in n-hexane, DCM, and EtOAc, respectively (Figure 4). A similar trend was observed in the bark extract of *M. longifolia*, with the highest FRAP values recorded in the MeOH (70.35 ± 6.32µmol Fe(II) equivalents/g dry extract) fraction, followed by the aqueous (40.23 ± 5.09µmol Fe(II) equivalents/g dry extract) fraction. However, the bark fractions exhibited lower FRAP activity than the corresponding leaf fractions. Moreover, the n-hexane, DCM, and EtOAc fractions of the bark extract exhibited comparatively lower FRAP activity, with values of 27.29 ± 4.51, 20.64 ± 3.61, and 35.52 ± 4.43 µmol Fe(II) equivalents/g dry extract, respectively.

### 2.5. α-Amylase Inhibitory Potential and Enzyme Kinetics Study of M. longifolia Leaf Extract

**Figure 5:**
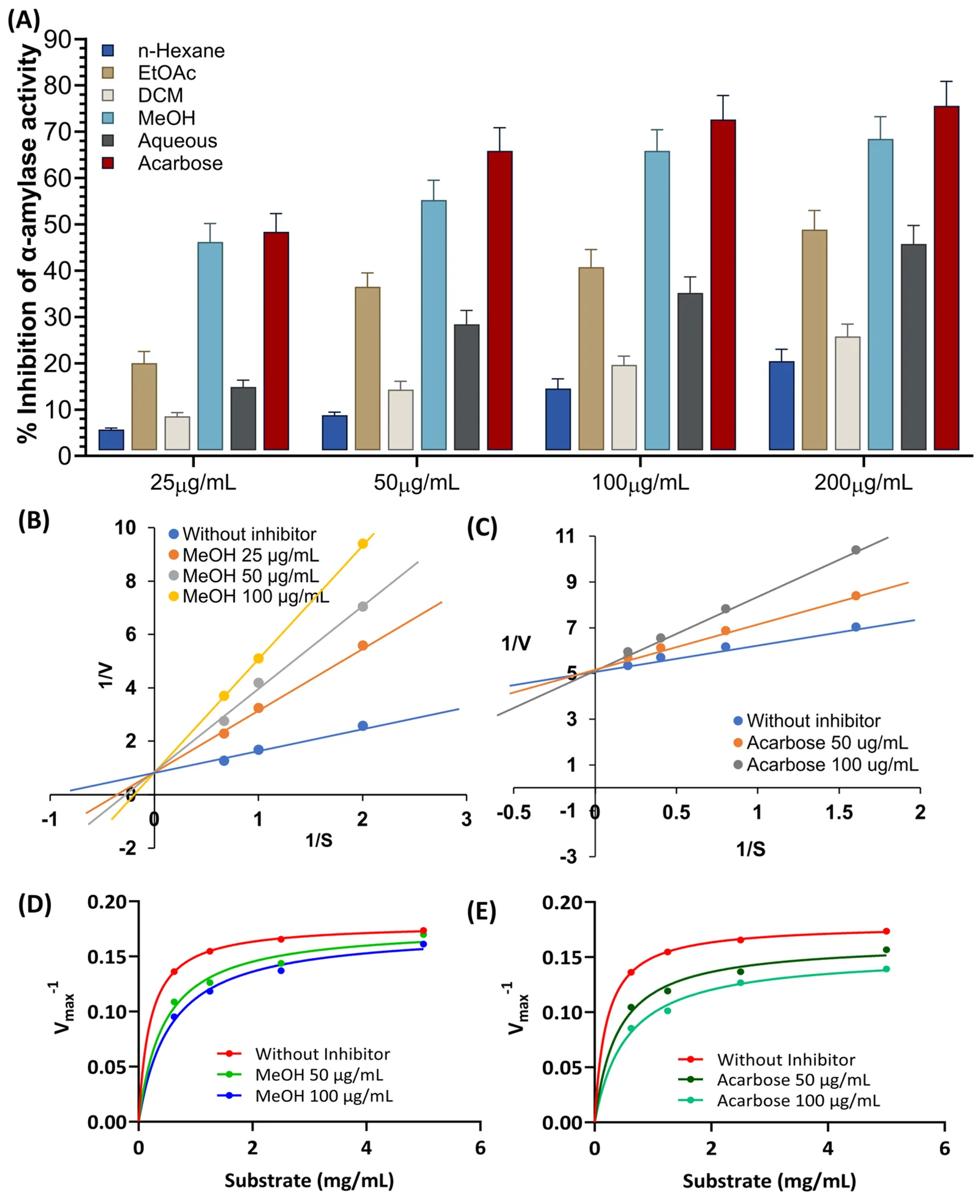
In vitro α-amylase inhibitory activity of (**A**): n-hexane, DCM, EtOAc, MeOH, and water fractions of *M. longifolia* leaf extract and reference drug acarbose. Values are expressed as mean ± SEM of triplicate (*n* = 3) assays from a single extraction batch. Kinetic studies of the *M. longifolia* leaf MeOH fraction-mediated mode of α-amylase inhibition. Lineweaver–Burk double reciprocal plot analysis of 1/V versus 1/S of (**B**): MeOH extract of *M. longifolia* leaf and (**C**): reference drug acarbose, against α-amylase inhibition. Nonlinear Michaelis–Menten substrate–velocity curves for (**D**) the MeOH fraction of *M. longifolia* leaf extract and (**E**) acarbose at the indicated inhibitor concentrations.

Hyperglycemia is managed using several therapeutic strategies, including the inhibition of key enzymes. Hyperglycemia can be managed using several therapeutic strategies, including inhibition of carbohydrate-digesting enzymes. α-Amylase and α-glucosidase play important roles in the digestion of dietary carbohydrates, and their pharmacological inhibition can reduce the rate of carbohydrate digestion and help attenuate postprandial glucose excursions [10]. Persistent hyperglycemia promotes the development of secondary complications of diabetes, including diabetic retinopathy, nephropathy, neuropathy, and cardiovascular diseases [35]. Although synthetic inhibitors are widely used for glycemic control, their long-term use is associated with several adverse effects [36]. To overcome these, there is growing interest in exploring natural products as potential sources of alternative therapeutic agents. In the present study, *M. longifolia* leaf and bark extracts exhibited promising antioxidant activity. However, leaf extracts demonstrated superior free radical-scavenging potential. Based on these findings, we investigated the anti-diabetic potential of *M. longifolia* leaf extracts by targeting α-amylase. Among all the fractions of *M. longifolia* leaf extracts, the MeOH (60.45%) fraction exhibited the highest α-amylase inhibition, followed by the aqueous (45.72%) fraction (Figure 5A). In comparison, the reference standard (acarbose) showed approximately 72% inhibition. The MeOH fraction exhibited an IC_50_ value of 32.11 µg/mL (95% CI: 20.24–42.80 µg/mL), whereas acarbose showed an IC_50_ value of 22.76 µg/mL (95% CI: 11.84–32.13 µg/mL) (Table 3). These beneficial effects may be attributed to the presence of diverse secondary metabolites, including phenols and flavonoids, which may occupy the catalytic site of α-amylase and restrict its activity. Our findings are consistent with previously published reports demonstrating that α-amylase and α-glucosidase are more effectively inhibited by more polar solvent extracts [37,38]. Undoubtedly, standard drugs for α-amylase inhibition are more effective at lower concentrations, but their long-term intake causes adverse effects, including hepatotoxicity and nephrotoxicity. Among the tested fractions, the MeOH extract of *M. longifolia* leaves exhibited the highest α-amylase inhibitory activity, indicating its potential as a source of α-amylase inhibitory phytochemicals. Furthermore, the inhibition kinetics of the MeOH fraction of *M. longifolia* leaf extract and acarbose were evaluated using Lineweaver–Burk double-reciprocal plots and global nonlinear regression of the substrate–velocity data. Competitive, noncompetitive, uncompetitive, and mixed-inhibition models were compared using Global R^2^ and the corrected Akaike information criterion (AICc), together with the corresponding V_max_, K*m*, and Ki/αKi estimates (Figure 5B–E, Table 4). For the MeOH fraction of *M. longifolia* leaf extract, the competitive model showed the lowest AICc (−123.32), whereas the mixed model showed a slightly higher Global R^2^ (0.9641 versus 0.9632 for the competitive model). The Ki value for the competitive model was 44.93 (95% CI: 25.00–77.86). For acarbose, the mixed model showed both the lowest AICc (−121.87) and the highest Global R^2^ (0.9817), with a corresponding Ki/αKi value of 44.70 (95% CI: 22.25–94.35) [10,39,40]. Given the chemically complex nature of the MeOH fraction, the observed kinetic behavior cannot be attributed to a single constituent or specific molecular mechanism without further experimental validation.

### 2.6. Determination of Compounds via GC-MS Analysis

**Figure 6:**
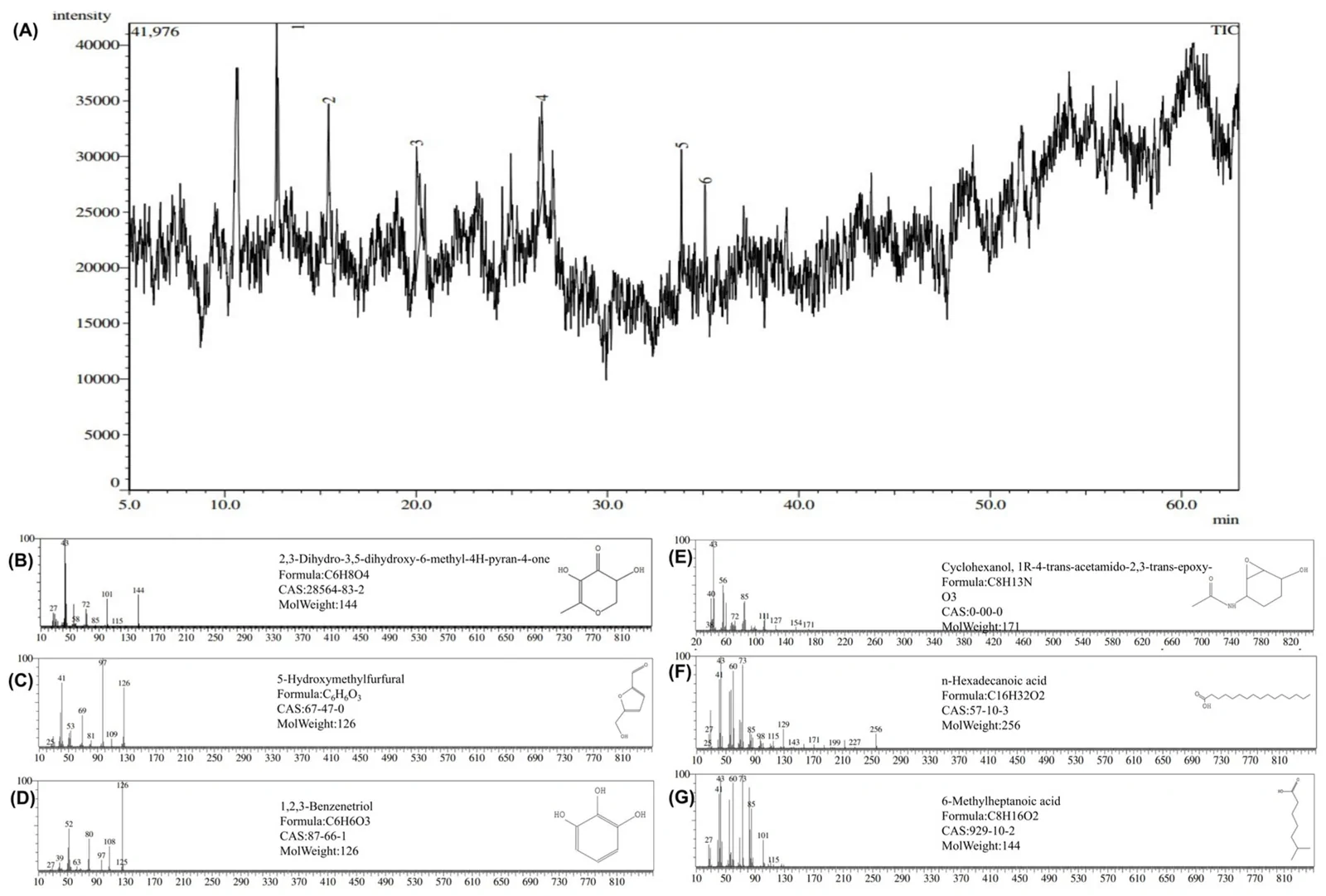
GC–MS analysis of the methanol (MeOH) leaf extract of *M. longifolia*. (**A**) Total ion chromatogram (TIC) of the MeOH leaf extract. (**B**–**G**) Chemical structures of the six putatively identified compounds.

The MeOH fraction of the leaf extract was subjected to GC–MS analysis to characterize its chemical constituents. The detected chromatographic peaks were evaluated by comparison with their mass spectra with reference spectra available in the NIST library, together with their observed retention times and relative peak areas. Based on these library spectral matches, six compounds were putatively identified: 4H-Pyran-4-one, 2,3-dihydro-3,5-dihydroxy-6-methyl-, 5-Hydroxymethylfurfural, 1,2,3-Benzenetriol, Cyclohexanol derivative (1R-4-trans-acetamido-2,3-trans-epoxy-), n-Hexadecanoic acid, and 6-Methylheptanoic acid (Table 5; Figure 6B–G). Representative chromatographic and mass spectral comparisons are provided in the Supplementary Material.

Although the leaf and bark fractions were comparatively evaluated for their biological activities, comprehensive chemical profiling of all fractions was not feasible because GC–MS analysis requires external analytical services and is constrained by the available research resources. Consequently, the six putatively identified compounds were identified only in the methanolic leaf fraction and could not be extrapolated to the chemical composition or biological activities of the other fractions.

### 2.7. Pharmacokinetic Profiling of GC-MS-Putatively Identified Compounds

The predicted physicochemical, drug-likeness, and pharmacokinetic properties of the compounds detected in the MeOH fraction of the leaf extract were evaluated using Swiss-ADME and PreADMET. SwissADME was used to assess the physicochemical and drug-like properties, whereas PreADMET was used to predict aqueous solubility, BBB permeation, Caco-2 and MDCK permeability, skin permeability (LogKp), plasma protein binding (PPB), human intestinal absorption (HIA), P-glycoprotein (P-gp) inhibition, CYP2D6 inhibition, and substrate status. The results are presented in Table 6 and Table 7, respectively. Furthermore, we evaluated the drug-likeness properties of the compounds according to Lipinski’s rule of five [41]. Lipinski’s rule states that a compound is considered “drug-like” if it meets the following criteria: molecular weight < 500 Da, hydrogen bond acceptors (HBA) not >10, hydroge n bond donors (HBD) not <5, and the partition coefficient (Log P) value of the octanol-water not more than 5 [42]. Importantly, the fulfillment of Lipinski’s rule of five indicates drug-like physicochemical characteristics but does not establish a compound as a viable drug candidate or demonstrate therapeutic efficacy. Our results showed that all compounds achieved satisfactory scores according to Lipinski’s rule of five (Table 6). Topological polar surface area (TPSA) and Log P are important physicochemical parameters related to molecular polarity and lipophilicity, and can provide preliminary information relevant to oral drug development. TPSA reflects molecular polarity and influences membrane permeability, whereas Log P indicates lipophilicity [43,44]. Generally, TPSA ≤ 60 Å^2^ indicates excellent intestinal absorption, whereas TPSA > 140 Å^2^ suggests poor membrane permeability. In the present study, 2,3-Dihydro-3,5-dihydroxy-6-methyl-4H-pyran-4-one and cyclohexanol, 1R-4-trans-acetamido-2,3-trans-epoxy- had TPSA values of 66.76 and 61.86, respectively. Although these values are slightly higher than the threshold, this single violation is insufficient to exclude these compounds from further investigation. However, the other identified compounds had acceptable Log P values (≤5). These physicochemical properties provide preliminary information supporting further pharmacological investigation of the identified compounds.

### 2.8. ADME Properties of Detected Compounds

The evaluation of absorption, distribution, metabolism, and excretion (ADME) properties using computational tools provides preliminary information on the pharmacokinetic behavior and physicochemical characteristics of bioactive compounds during early stage drug discovery [45]. Our ADME analysis demonstrated that all compounds had acceptable aqueous solubility (Table 6). Moreover, all compounds showed blood–brain barrier (BBB) permeability scores within the recommended threshold value, except n-hexadecanoic acid, which exhibited a BBB score of 8.21, exceeding the acceptable limit of 2. According to the established BBB permeability score, >2.0 is considered high, 2.0–0.1 (moderate), and <0.1 indicates low infiltration [46,47]. Additionally, determining the permeability of MDCK and Caco-2 cells is an important step in drug discovery [48]. The PreADMET analysis predicted Caco-2 cell permeability values ranging from 1.8 to 26.07 nm/s and MDCK cell permeability values ranging from 1.24 to 152.57 nm/s. According to the established classification, permeability values of <4, 4–70, and >70 nm/s represent low, moderate, and high membrane permeability, respectively [49]. The predicted Caco-2 and MDCK permeability values varied among the evaluated compounds, indicating differences in their predicted epithelial permeability using the applied computational model. Furthermore, we evaluated skin permeability (Log Kp), which demonstrates the rate at which a chemical entity penetrates the outermost layer of the skin into the systemic circulation [50]. The Log Kp values ranging from −0.58 to −5.00 cm/h were considered satisfactory [51]. The results indicated that the Log Kp values of all the GC-MS-putatively identified compounds fell within the acceptable range, suggesting favorable skin permeability characteristics (Table 7).

Several factors, including lipophilicity, molecular size, molecular weight, ionization, specialized barriers, and capillary permeability, influence drug distribution from plasma to target tissues. Plasma protein binding (PPB) percentage measures the proportion of a drug that attaches to the protein in the blood. Only the unbound fraction of the drug is permeable to the cell membrane, reaches the site of action, and performs pharmacological action. In drug design, the ideal therapeutic benchmark and threshold for PPB include the following considerations: the computational PPB threshold value should be ≤90%. A chemical entity with a PPB > 90% is considered highly protein-bound and can act as a drug reservoir and prolong half-life [52,53]. Our results revealed that the PPB values of 1, 2, 3-benzenetriol, n-hexadecanoic acid, and 6-methylheptanoic acid were 100%, whereas 2,3-Dihydro-3,5-dihydroxy-6-methyl-4H-pyran-4-one, 5-hydroxymethylfurfural, and cyclohexanol, 1R-4-trans-acetamido-2,3-trans-epoxy- were 38.43, 49.46, and 16.57%, respectively. Based on the predicted PPB values, compounds with PPB values below 90% may exhibit a greater unbound fraction in plasma than those with PPB values of 100%.

In contrast, the absorption, bioavailability, and excretion of drugs depend on human intestinal absorption (HIA) [54]. The predicted HIA values for the evaluated compounds ranged from 75.91% to 98.29%, indicating a predicted capacity for intestinal absorption using the applied computational model. These predictions provide preliminary information on intestinal permeability and should not be interpreted as direct evidence of systemic bioavailability or pharmacological efficacy of the compounds. The reference drug, acarbose, exhibited a predicted HIA value of 0.00%. This value should be interpreted in the context of the pharmacological action of acarbose, which is primarily localized within the gastrointestinal tract; therefore, low predicted systemic absorption should not be considered an intrinsic disadvantage of this drug. CYP2D6 is an important drug-metabolizing enzyme involved in the metabolism of approximately 20–25% of commonly used drugs [55,56]. CYP2D6-mediated drug metabolism varies among individuals, with some being rapid metabolizers and others being poor metabolizers. CYP2D6 metabolizer status can influence drug response and toxicity; for example, poor metabolizers may exhibit reduced efficacy of CYP2D6-dependent prodrugs, whereas ultra-rapid metabolizers may be at increased risk of toxicity [57]. Consequently, CYP2D6 plays a key role in dose optimization and personalized pharmacotherapy [56]. The predicted CYP2D6-related profiles differed among the evaluated compounds (Table 7). Cyclohexanol, 1R-4-trans-acetamido-2,3-trans-epoxy- (PubChem CID: 537214) was predicted to be an inhibitor of CYP2D6, whereas the remaining compounds, including n-hexadecanoic acid (PubChem CID: 985), were predicted to be P-gp inhibitors and non-inhibitors of CYP2D6. These computational predictions provide preliminary information on potential CYP2D6 interactions and should not be interpreted as experimental evidence of enzyme inhibition or altered drug metabolism.

### 2.9. In Silico Toxicity Prediction of GC-MS-Putatively Identified Compounds

The predicted toxicity profiles of the GC-MS-putatively identified compounds were evaluated using ProTox 3.0 (Table 8). The predicted acute oral LD_50_ values ranged from 300 to 5000 mg/kg, corresponding to toxicity classes III to V. Most compounds were predicted to be inactive for the evaluated toxicity endpoints, although active predictions were observed for some organ toxicities and toxicological endpoints. These findings provide preliminary computational indications of potential toxicity liabilities but should not be interpreted as direct evidence of toxicity or safety of the compounds. However, experimental toxicological studies are required to validate these predictions.

### 2.10. Molecular Docking of GC-MS-Putatively Identified Compounds with α-Amylase

**Figure 7:**
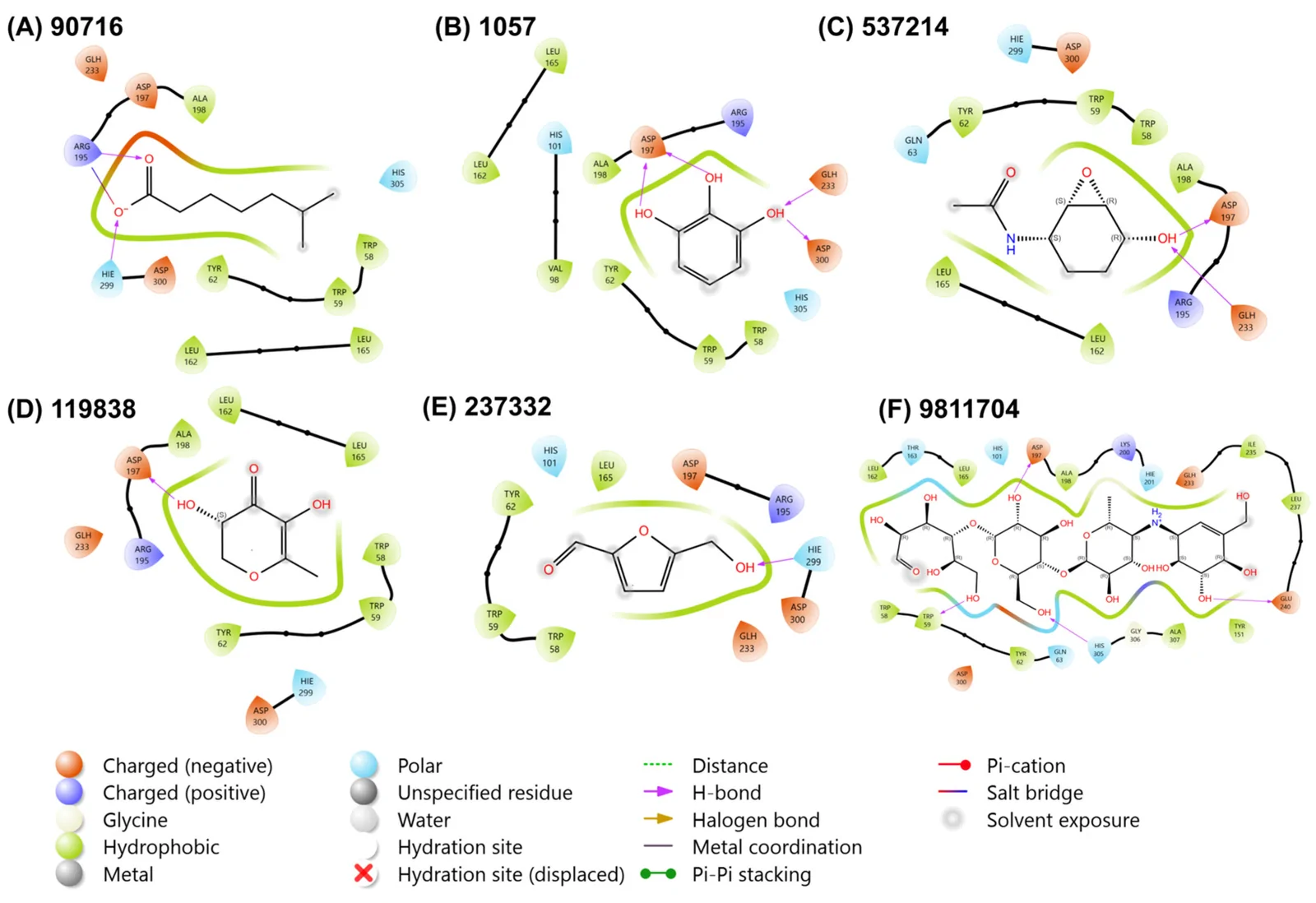
In silico binding patterns of GC-MS-putatively identified compounds against the active pocket of α-amylase (PDB ID: 3BAJ). **Note:** Glh: Glu; Hie: His. The 2D line diagrams represent best docking complexes and their interacting residues of α-amylase active pocket with, (**A**) 6-methylheptanoic acid (PubChem CID: 90716), (**B**) 1, 2, 3-benzenetriol (PubChem CID:1057), (**C**) Cyclohexanol, 1R-4-trans-acetamido-2,3-trans-epoxy- (PubChem CID:537214), (**D**) 2,3-Dihydro-3,5-dihydroxy-6-methyl-4H-pyran-4-one (PubChem CID:119838), (**E**) 5-hydroxymethylfurfural (PubChem CID:237332), (**F**) and standard drug acarbose (PubChem CID:9811704).

The GC-MS-putatively identified compounds docked within the active pocket of α-amylase. Computational molecular docking analysis is widely used to investigate the predicted interactions and relative scoring of chemical entities with their target proteins [10,58,59]. Similarly, our in silico molecular docking analysis was performed to investigate the predicted interactions of the identified compounds with the binding site of α-amylase. The identified compounds were predicted to interact with the catalytic site of α-amylase. The docking scores ranged from −2.205 to −6.127, while the corresponding Glide scores ranged from −2.210 to −6.132 (Table 9). Among the identified compounds, 6-methylheptanoic acid (PubChem CID: 90716) showed the most negative Glide score (−6.132). This interaction was stabilized by 12 amino acids (Trp58, Trp59, Tyr62, Leu162, Leu165, Arg195, Asp197, Ala198, Glu233, His299, Asp300, His305.) with two H-bond interaction (Arg195, and His299) and one salt bridge (Arg195). Similarly, 1,2,3-Benzenetriol (PubChem CID: 1057) stabilized the complex structure by 13 amino acid residues (Trp58, Trp59, Tyr62, Val98, His101, Leu162, Leu165, Arg195, Asp197, Ala198, Glu233, Asp300, His305), four hydrogen bonds were formed with three residues (Asp197, Glu233, and Asp300), with Asp197 contributing two hydrogen bonds, while Glu233 and Asp300 contributed one hydrogen bond each, with a Glide score of −4.754 (Figure 7, Table 9). Cyclohexanol, 1R-4-trans-acetamido-2,3-trans-epoxy- (PubChem CID: 537214), 2,3-dihydro-3,5-dihydroxy-6-methyl-4H-pyran-4-one (PubChem CID: 119838), hydroxymethylfurfural (PubChem CID: 237332), and n-hexadecanoic acid (PubChem CID: 985) showed Glide scores of −3.761, −4.449, −2.843, and −2.210, respectively (Table 9). In contrast, acarbose (PubChem CID:9811704), occupied the active site of α-amylase by 21 amino acid residues (i.e., Trp58, Trp59, Tyr62, Gln63, His101, Tyr151, Thr163, Leu162, Leu165, Asp197, Ala198, Lys200, His201, Glu233, Ile235, Leu237, Glu240, Asp300, His305, Gly306, Ala307.) and 4-H bonds (Trp59, Asp197, Glu240, His305). Interestingly, all the compounds stabilized the complex structure with almost similar amino acid residues compared to the standard (acarbose). The Glide score of 6-methylheptanoic acid was very similar to that of acarbose (−6.132 and −6.120, respectively), indicating comparable predicted docking scores under the applied docking conditions. These findings identify 6-methylheptanoic acid as a preliminary α-amylase-interacting lead for further investigation; however, experimental studies using the purified individual compound are required to confirm its inhibitory activity and mechanism.

A limitation of the present study is that the in vitro α-amylase inhibition assay was performed using porcine pancreatic α-amylase, whereas the molecular docking analysis was conducted using human pancreatic α-amylase (PDB ID: 3BAJ). The human α-amylase structure was selected because a structurally characterized crystal structure with a co-crystallized inhibitor was available for docking and protocol validation. Therefore, the docking results provide exploratory structural insights into the potential interactions of the identified compounds with human α-amylase but should not be interpreted as direct mechanistic evidence for the inhibition observed with the porcine enzyme. Moreover, the docking of GC–MS-putatively identified compounds does not establish that these individual compounds are responsible for the α-amylase inhibitory activity of the crude extract. In particular, the potential contribution of 6-methylheptanoic acid requires experimental validation using the purified individual compound.

A limitation of the present study is the incomplete archival documentation of plant collections and botanical authentication, including the absence of a documented voucher specimen/accession number. Future investigations should incorporate formal botanical authentication and voucher deposition, together with comprehensive collection records, to strengthen reproducibility. No cytotoxicity or selectivity assessment was performed in the present study; therefore, the safety and selectivity of the extracts or their identified constituents cannot be inferred from the current findings and require dedicated experimental evaluation.

## 3. Materials and Methods

### 3.1. Chemical and Reagents

All the solvents, n-hexane, dichloromethane (DCM), ethyl acetate (EtOAc), and methanol (MeOH), were purchased from Thermo Fisher Scientific Pvt. Ltd., MA/USA. The ascorbic acid, 1,1-diphenyl-2-picrylhydrazyl (DPPH), 2,4,6-tripyridyl-s-triazine (TPTZ), ferrous sulphate, and ferric chloride, Dinitro-salicylic acid (DNS), Sucrose, and starch were procured from Hi Media laboratories Pvt. Ltd., Mumbai/India. The standard acarbose drug and pancreatic α-amylase were obtained from Sigma-Aldrich, Burlington, MA/USA, and Sisco Research Lab. Pvt. Ltd. (SRL), New Delhi, India, respectively. The remaining reagents utilized in this study were analytical grade.

### 3.2. Collection, Processing, and Preparation of Plant Material for Soxhlet-Based Extraction

The *M. longifolia* leaves and bark were collected in February 2023 from the surroundings of Integral University, Lucknow, India. The collected plant material was identified as *M. longifolia*. Extraction of the leaf and bark was performed according to a previously published protocol [9]. Briefly, fresh green leaves and bark of *M. longifolia* were cleaned with double-distilled water to eliminate dust and dried at room temperature for 4–6 days, avoiding sun-drying because it may cause substantial changes in bioactive chemicals. Dehydrated leaf and bark were crushed in a grinder to obtain fine powder. A total of 50 g of leaf and bark powder was taken separately for extraction in a Soxhlet apparatus using non-polar to polar solvents sequentially (n-hexane > dichloromethane (DCM) > ethyl acetate (EtOAc) > methanol (MeOH) > double distilled water (dH_2_O) until the solvent became colorless or for a minimum of 24 h for each solvent. At the initial stage, 500 mL of each solvent was used, and additional solvent was added when required during extraction. The extraction temperature was maintained at the boiling point of the respective solvent. Following extraction with each solvent, the solvent containing the extract was transferred to large Petri dishes and allowed to evaporate at room temperature until completely dry. The plant residue remaining after each extraction was then dried overnight at room temperature before proceeding with extraction using the next solvent. This procedure was repeated sequentially for all five solvents. After completion of the sequential extraction, the dried extracts were collected separately in Eppendorf tubes, weighed, and stored at −20 °C until further analysis. The percentage yield of sequentially extracted plant extracts in different solvent systems was evaluated using the following formula:

%*Yield* = (weight of crude extract)/(weight of raw material) × 100

### 3.3. Qualitative Phytochemical Analysis of M. longifolia Plant Extract

The leaf and bark fractions of *M. longifolia* were qualitatively examined for phytoconstituents to identify the major classes of secondary metabolites, including tannins, flavonoids, phenols, alkaloids, saponins, steroids, triterpenoids, and cardiac glycosides, according to previously published protocols by Matseke et al. [60].

### 3.4. DPPH Radical Scavenging Potential of M. longifolia Plant Extract

Each dried extract was dissolved in 100% DMSO and then diluted with assay buffer to create stock solutions of the dried extracts (n-hexane, DCM, and EtOAc) and different working concentrations (5, 10, 15, 20, 25, and 30 µg/mL) through serial dilution. For each stock, the final DMSO concentration was maintained at <1% (*v*/*v*), a level that was shown to have no scavenging effect on DPPH or ABTS radicals in the vehicle-only control. The DPPH radical-scavenging activity was determined according to the method of Brand-Williams et al. [61], with modifications based on the procedure described by Alvi et al. [62]. Briefly, 100 µL of varied concentrations (5, 10, 15, 20, 25, and 30 μg/mL prepared by serial dilution) of *M. longifolia* leaf or bark extracts or standard (ascorbic acid) was added to 2.9 mL of DPPH solution and placed in the dark at room temperature for 30 min. Each assay was carried out in technical triplicates (*n* = 3 reactions) from a single extract preparation rather than independent biological or extraction replicates, to assess analytical measurement precision. The absorbance was recorded at 517 nm. The violet color diminished together with the generation of the reduced form of DPPH. The percentage (%) of DPPH-free radical scavenging activity was computed using the following formula:

% DPPH radical scavenging = (Abs.of Control − Abs.of test sample)/(*Abs*.*of control*) × 100

A gradient concentration range (5 to 30 µg/mL) was used to assess the radical-scavenging capabilities against DPPH. The IC_50_, which is presented as concentration in µg/mL, was calculated using non-linear regression analysis of the dose–response curve.

### 3.5. Ferric Reducing Antioxidant Potential

Benzie and Strain’s [63] protocol was implemented with slight modifications to evaluate the ferric reducing antioxidant power (FRAP) of *M. longifolia* leaf and bark fractions [64]. Briefly, FRAP working solution was prepared by mixing sodium acetate buffer (300 mM, pH 3.6); TPTZ (10 mM) prepared in 40 mM HCl; and FeCl_3_ (20 mM) in a ratio of 10:1:1, respectively. Then, 2.9 mL of FRAP working solution was added to 100 µL of various concentrations of *M. longifolia* leaf and bark extracts, vortexed, and incubated at 37 °C for 30 min, and the optical density was recorded at 593 nm. The standard curve of FeSO_4_ was used to calculate the results, which are expressed as μmol Fe (II) equivalents per gram of dried extract/fraction (μmol Fe(II)/g dry extract) of *M. longifolia* leaf or bark extract powder.

### 3.6. ABTS Radical Scavenging Activity

The ABTS solution was prepared by mixing 7 mM ABTS with 2.45 mM potassium persulfate. The solution was incubated overnight in the dark to allow for stable radical formation. Before the experiment, the solution was diluted to obtain an absorbance of 0.70 at 734 nm. Thereafter, 100 μL of each extract at concentrations of 5, 10, 15, 20, 25, and 30 μg/mL, prepared by serial dilution, was added to 900 μL of ABTS solution and incubated in the dark for 30 min at 37 °C. The absorbance was recorded at 734 nm using an Eppendorf Biospectrometer Kinetics (Eppendorf, Hamburg/Germany) [65]. The percentage of ABTS radical scavenging was calculated using the following equation:

% *ABTS radical scavenging* = (Abs.of control − Abs.of test)/(Abs.of control) × 100

The radical-scavenging activities against ABTS were evaluated across a concentration gradient range (5–30 µg/mL). The IC_50_, presented as a concentration in µg/mL, was calculated using non-linear regression analysis of the dose–response curve.

### 3.7. α-Amylase Inhibitory Assay

The in vitro inhibiting potential of *α*-amylase by *M. longifolia* leaf extract (n-hexane, EtOAc, DCM, MeOH and aqueous) was evaluated according to published and standardized method [9,10]. In order to assess the enzyme activity, reaction linearity, incubation time, and enzyme concentration, we adapted a previously published and established protocol [10]. Briefly, in 20 mM PBS (pH 6.7) supplemented with 6.7 mM NaCl, porcine pancreatic α-amylase (0.15 units/mL) and soluble potato starch substrate (0.5% w/v) were pre-pared. The enzyme concentration (0.15 U/mL) and reaction incubation time (15 min at 37 °C) were experimentally pre-validated in preliminary time-course and enzyme-dilution trials to ensure steady-state conditions within the linear velocity phase (A540 ~0.2–0.8; <10% total substrate conversion) before DNS reagent addition. Followed by the main experiment. 0.15 units/mL of *α*-amylase (porcine pancreatic) was prepared in chilled PBS (20 mM, pH 6.7), containing 6.7 mM NaCl. 250 µL of α-amylase solution was added to 100 µL of each *M. longifolia* leaf extract fraction (n-hexane, DCM, EtOAc, MeOH, and aqueous), except for the corresponding blank, and preincubated at 37 °C for 20 min. The extracts were evaluated at concentrations of 25, 50, 100, and 200 µg/mL. To start the reaction 250 µL of 0.5% starch (substrate), prepared in PBS (20 mM, pH 6.7), was added and vortexed. Following the incubation for 15 min at 37 °C, 2 mL of DNS solution (40 mM), containing NaOH (0.4 M), and K-Na tartrate (1 M) was added and vortexed. The reaction mixture was placed in a water bath boiled at 100 °C for 10 min. After that the reaction mixture was immediately cooled down and absorbance was recorded at 540 nm. The experiment was performed in triplicate. Acarbose was used as a reference standard. To calculate the % inhibition of α-amylase activity, the following formula was used:

% *Inhibition* = 100−% *reaction*

where % reaction = (mean product in sample/mean product in control) × 100. The IC_50_ values for α-amylase inhibition were determined by non-linear concentration–response regression using a variable-slope model, and the corresponding 95% confidence intervals were estimated by profile likelihood.

Sample blank tubes containing each extract and starch substrate, but without enzyme, were subjected to the same incubation and boiling conditions to account for intrinsic extract absorbance and non-enzymatic reduction in DNS. The background-corrected absorbance was used to calculate α-amylase inhibition.

### 3.8. Determination of Mode of Action (Kinetics of α-Amylase)

In order to elucidate the mechanism by which *M. longifolia* (MeOH leaf extract) inhibits *α*-amylase activity, the kinetics study was performed according to the previously published method [9]. Briefly, to conduct the experiment, 2 sets (A: in the presence and B: absence of inhibitor) of reactions were carried out to evaluate enzyme activity. For the kinetic inhibition experiments, the MeOH leaf fraction was evaluated at 50, and 100 µg/mL, whereas acarbose was evaluated at 50 and 100 µg/mL. The inhibitor was added to the reaction mixture before addition of α-amylase, and the corresponding uninhibited control contained PBS in place of the inhibitor. Then 250 µL of 0.15 units/mL *α*-amylase was added. While in B set: 100 µL of PBS (20 mM, pH 6.7 with NaCl (6.7 mM) in place of the inhibitor or test sample was added in each reaction mixture. Both sets of reaction mixture were thoroughly stirred and pre-incubated in a water bath at 37 °C for 20 min. Then 100 µL of varied concentrations of substrate ranging from 5 mg/mL to 0.156 mg/mL (prepared by serial dilution) was added in each reaction of both sets. Following the incubation for 15 min at 37 °C, 2 mL DNS solution was added and incubated at 100 °C for 10 min in a water bath. The reaction mixture was cooled down and absorbance was taken at 540 nm. The inhibition kinetics of the MeOH fraction of *M. longifolia* leaf extract and acarbose were evaluated using Lineweaver–Burk double-reciprocal plots and global nonlinear regression of the substrate–velocity data. Competitive, noncompetitive, uncompetitive, and mixed-inhibition models were fitted and compared using Global R^2^ and the corrected Akaike information criterion (AICc). The corresponding V_max_, K*m*, and Ki/αKi values with 95% confidence intervals (CI) were estimated for each model. Lower AICc and higher Global R^2^ values were considered indicative of better model fit.

### 3.9. Data Analysis

All experimental assays were performed in triplicate (*n* = 3 reactions) from a single extraction batch, and values are expressed descriptively as mean ± SEM. For intrinsic sample pigmentation and to eliminate spectral interference at the measurement wavelengths (for DPPH, ABTS, and FRAP), extract blanks were prepared in parallel across all tested concentrations (5–30 µg/mL). These blanks contained 100 µL of extract diluted in assay buffer/solvent without the chromogenic radical or reagent solutions. The net absorbance was observed by subtracting the background OD of the extract blank from the reaction absorbance:

A*bsorbance*_*Final*_ = *Absorbance_Reaction_* − *Absorbance_Extract_*_*blank*_

The IC_50_ values for the DPPH and ABTS radical-scavenging assays were determined by nonlinear concentration–response regression using a variable-slope model in GraphPad Prism 10, with 95% confidence intervals estimated by profile likelihood. Formal inferential statistical hypothesis testing (such as ANOVA or post hoc pairwise comparisons) was not conducted; consequently, no claims of statistical significance were made, and the data were interpreted purely descriptively.

### 3.10. Gas Chromatography-Mass Spectrometry (GC-MS) Analysis

The chemical constituents of the MeOH leaf extract were identified using an GCMS-QP2010 Ultra (Shimadzu Corporation, Kyoto, Japan) instrument. Helium gas was used as carrier gas at 1 mL/min column velocity flow. The interface and ion-source temperatures were maintained at 300 °C and 250 °C, respectively, with a pressure of 16.2 psi. A 1 μL aliquot of the sample was injected in a split mode using a split ratio of 1:50. The injection temperature was maintained at 300 °C with a solvent delay of 1.8 min. The temperature of the column was first maintained at 36 °C for 5 min, then it was progressively raised to 150 °C at a rate of 4 °C per min^−1^. Afterwards, it was raised to 250 °C at a rate of 20 °C min^−1^ and maintained for 5 min. The relative percentage value of each component was assessed by comparing its average peak area to the total peak area. The detected compounds were putatively identified by comparing their acquired mass spectra with reference spectra available in the National Institute of Standards and Technology (NIST) library, together with their chromatographic retention times and relative peak areas. Because library-based spectral matching alone does not provide unequivocal structural confirmation, the reported compound assignments were considered putative in the absence of authentic reference standards, experimentally determined retention indices, or orthogonal analytical confirmation.

### 3.11. ADME and Drug-Likeness Studies of Selected Ligands

The SMILES structures of the six GC-MS-putatively identified compounds were obtained from PubChem and used as input structures for in silico ADME analyses. SwissADME (http://www.swissadme.ch, accessed on 13 February 2026) was used to evaluate the physicochemical and drug-like properties, including heavy atoms, rotatable bonds, hydrogen-bond acceptors, hydrogen-bond donors, molar refractivity, topological polar surface area (TPSA), iLogP, and ESOL-based aqueous solubility and solubility class. PreADMET (https://preadmet.webservice.bmdrc.org/, accessed on 13 February 2026) was used to predict absorption-, distribution-, and permeability-related endpoints, including aqueous solubility, blood–brain barrier (BBB) permeation, Caco-2 and MDCK permeability, skin permeability (LogKp), plasma protein binding (PPB), human intestinal absorption (HIA), P-glycoprotein (P-gp) inhibition, CYP2D6 inhibition, and substrate status. The input SMILES structures are provided in the Supplementary Material (Table S1), and the corresponding predicted endpoints and units are reported in Table 7.

### 3.12. In Silico Toxicity Prediction

To determine the computational toxicity endpoints, the canonical SMILES of all GC-MS-putatively identified compounds were submitted to the ProTox server 3.0. The predicted endpoints included acute oral toxicity (LD_50_ in mg/kg and toxicity classification from Class I to VI), organ toxicity (hepatotoxicity), and toxicological hazards (mutagenicity, carcinogenicity, cytotoxicity, and immuno-toxicity), accompanied by model prediction confidence scores [66].

### 3.13. Retrieval and Structure Preparation of the 3D-Structure of the Ligand and Target Protein

The GC-MS-putatively identified 3D structures of compounds were obtained in .sdf file format from the PubChem Database (http://pubchem.ncbi.nlm.nih.gov, accessed on 13 February 2026) and integrated into the Maestro workspace. To perform the molecular docking studies, ligand structure preparation and minimization were completed using the LigPrep module, selecting the OPLS4 force field. The Epik module was used to explore the potential ionization and tautomerization of selected compounds at pH 7.0 ± 2.0 [67]. Moreover, the 3D structure of α-amylase (human) (PDB ID: 3BAJ) obtained from the protein data bank (PDB) (http://www.rcsb.org/, accessed on 16 February 2026) and incorporated in Maestro 13.9 via the ‘Get PDB’ option. The protein was prepared using the ‘protein preparation’ wizards from the Maestro module of Schrödinger’s suite. The target protein was preprocessed with Prime, involving elimination of water beyond 5 Å from the het group, assignment of hydrogen atoms, bond ordering, conversion of selenomethionines to methionines, and OPLS4 force field for minimization, as well as filled missing loops and side chains at pH 7.0 ± 2.0 via selecting the Epik tool [68] to achieve a structural root mean square deviation (RMSD) of 0.30 for all heavy atoms during protein preparation. The following software and tools, i.e., Schrödinger Suite (2025-1), Discovery Studio Visualizer 2021, ChemDraw Professional version 18.1, and Notepad^++^ v7.9, were used for the preparation of the docking file and post-visualization. To provide preliminary structural insights on possible ligand-target interactions pertinent to human physiology, the molecular docking analysis was conducted against the crystal structure of human pancreatic α-amylase (3BAJ). Although porcine pancreatic α-amylase was used as a standard enzymatic model in in vitro inhibition assays, human pancreatic α-amylase shows a high overall sequence identity (~87%) and strict conservation of important active site catalytic residues (Asp197, Glu233, and Asp300) with porcine pancreatic α-amylase, offering a reasonable structural surrogate. On the other hand, docking simulations were only considered predictive and hypothesis-generating.

### 3.14. Active Site Prediction, Receptor Grid Generation, and Molecular Docking Studies

Potential binding sites of α-amylase (PDB ID: 3BAJ) were identified using Schrödinger’s SiteMap tool with default parameters. The top-ranked site based on the SiteScore and corresponding site volume was selected for receptor-grid generation [69]. The maximum site score and volume size were selected to create the grid for the receptor to dock. The grid was created on the active pocket using the default parameters of Van der Waals radius scaling of 1.0 with a cut-off of 0.25 partial charges. Moreover, the target protein (3BAJ) complexed with a native crystallographic ligand, bound to its identical binding site (earlier observed in SiteMap). Both constructed binding sites via SiteMap and co-crystallized ligand with 3BAJ exhibited identical binding pockets. Therefore, through the ‘Receptor-Grid-Generation tool’, the “pick to identify the ligand molecule” option was selected to generate a receptor grid using the default settings in the Glide module to specify the binding site. For the docking studies, the ‘Protein-ligand docking’ tools from the Glide module were employed, with the extra-precision (XP) method, and the job was submitted using default settings. The docking protocol was validated by redocking the co-crystallized acarbose into the binding site of α-amylase (PDB ID: 3BAJ). The crystallographic acarbose was removed from the protein structure before redocking, and the same ligand preparation and Glide XP docking workflow used for the selected GC-MS-putatively identified compounds was applied. The receptor grid was centered at X = 10.15, Y = 15.84, and Z = 41.12 Å, with a grid box dimension of 20 × 20 × 20 Å. The redocked acarbose pose was superimposed with the experimentally observed crystallographic pose, and the heavy-atom root mean square deviation (RMSD) was calculated. The redocking yielded a heavy-atom RMSD of 2.196 Å (Figure S1), which is slightly above the commonly used 2.0 Å criterion but indicates reasonable reproduction of the experimentally observed acarbose binding pose. After the completion of the molecular docking studies, the Maestro v. 13.9 tool was used to analyze the ligand binding interactions.

## 4. Conclusions

Based on the present findings, the MeOH fraction of *M. longifolia* leaf extract exhibited comparatively higher in vitro α-amylase inhibitory activity together with substantial antioxidant activity. Global nonlinear kinetic analysis indicated that the MeOH fraction of *M. longifolia* leaf extract was best described by a competitive inhibition model. However, because the MeOH fraction is a chemically complex crude extract, the observed kinetic behavior cannot be attributed to a single constituent or a specific molecular mechanism without further experimental validation. Additionally, this fraction also possesses strong DPPH and ABTS free radical scavenging potential, suggesting its use in oxidative stress management. These beneficial effects of the extract might be due to the presence of phytochemicals, including phenols and flavonoids. Furthermore, the six major compounds were putatively identified through GC-MS analysis of the MeOH fraction of *M. longifolia* leaf extract. Our in silico study suggested that these compounds may interact with the catalytic pocket of human α-amylase. Taken together, the findings provide preliminary in vitro evidence of antioxidant capacity and α-amylase inhibitory activity in the tested *M. longifolia* extracts. The observed activities should therefore be considered preliminary and require further investigation through quantitative chemical characterization, isolation, and validation of individual constituents, cellular and pharmacodynamic studies, experimental toxicity assessment, and in vivo efficacy evaluation.

## Figures and Tables

**Table 1:** Percentage extract yield of *M. longifolia* leaf and bark obtained using different solvents.

Extract Fraction	%Yield of Leaf Extract	%Yield of Bark Extract
n-hexane	3.51	1.83
DCM	4.62	3.19
EtOAc	7.12	3.76
MeOH	9.84	5.26
Aqueous	12.79	4.52

**Table 2:** Phytochemical screening of *M. longifolia* leaf and bark extracts.

Phytochemicals	*M. longifolia* Leaves Extract					*M. longifolia* Bark Extract				
	n-Hexane	DCM	EtOAc	MeOH	Aqueous	n-Hexane	DCM	EtOAc	MeOH	Aqueous
Tannins	−	+	−	+	+	−	−	+	+	+
Saponins	−	+	−	−	+	−	+	+	+	+
Alkaloids	−	−	+	+	+	−	−	+	+	+
Flavonoids	−	−	+	+	+	+	−	+	+	+
Glycosides	+	−	−	+	+	−	−	+	+	+
Phenols	−	+	+	+	+	−	−	+	+	+
Terpenoids	+	+	+	+	−	+	+	−	+	−
Cardiac glycosides	−	−	−	−	−	−	−	+	−	+
Steroids	+	−	−	−	+	+	−	+	−	−

**Note:** − indicates absence and + indicates the presence of the tested phytochemical class.

**Table 3:** Calculated IC_50_ values of DPPH, ABTS, and α-amylase.

**DPPH**	**Leaf Fractions**		
	**Fraction**	**IC_50_ (μg/mL)**	**95% CI**
	MeOH	8.67	7.178–10.10
	Aqueous	10.51	9.166–11.85
	**Bark Fractions**		
	MeOH	8.21	7.660–8.788
	Aqueous	8.15	6.822–9.447
	Ascorbic Acid	13.70	11.76–15.64
**ABTS**	**Leaf Fractions**		
	DCM	4.82	4.445–5.219
	EtOAc	9.38	8.319–10.47
	MeOH	4.32	4.005–4.641
	Aqueous	4.68	4.299–5.081
	**Bark Fractions**		
	MeOH	4.44	3.930–4.870
	Aqueous	5.40	4.620–6.136
	Ascorbic Acid	10.81	9.457–12.22
**α-Amylase inhibition**	Leaf-MeOH	32.11	20.24–42.80
	Acarbose	22.76	11.84–32.13

**Table 4:** Comparison of α-amylase inhibition models using global nonlinear regression.

Sample	Inhibition Model	V_max_ (95% CI)	K*m* (95%CI)	Ki/αKi (95% CI)	AICc	Global R^2^
Acarbose	Competitive	0.1695 (0.1594–0.1804)	0.1323 (0.04628–0.2345)	20.63 (7.130–40.29)	−114.92	0.9516
	Noncompetitive	0.1910 (0.1768–0.2063)	0.3275 (0.2145–0.4629)	260.9 (195.2–377.1)	−111.84	0.9375
	Uncompetitive	0.1925 (0.1735–0.2141)	0.3577 (0.1995–0.5597)	227.4 (156.2–378.8)	−106.37	0.9014
	Mixed-Model	0.1793 (0.1698–0.1895)	0.2014 (0.1228–0.2919)	44.70 (22.25–94.35)	−121.87	0.9817
Leaf MeOH fraction	Competitive	0.1779 (0.1697–0.1865)	0.1957 (0.1192–0.2833)	44.93 (25.00–77.86)	−123.32	0.9632
	Noncompetitive	0.1931 (0.1767–0.2110)	0.3584 (0.2272–0.5198)	451.7 (285.6–953.2)	−120.62	0.8995
	Uncompetitive	0.1940 (0.1742–0.2164)	0.3836 (0.2217–0.5913)	405.0 (234.2–1122)	−110.70	0.8690
	Mixed-Model	0.1795 (0.1693–0.1870)	0.2080 (0.1158–0.2879)	50.96 (24.22–80.05)	−108.07	0.9641

**Table 5:** Putatively identified phytochemical constituents in the methanolic leaf extract of *M. longifolia*, as determined by GC–MS.

Retention Time	Area%	Compound Name	Chemical Formula	Mol. Weight
12.715	13.95	2,3-Dihydro-3,5-dihydroxy-6-methyl-4H-pyran-4-one	C_6_H_8_O_4_	144
15.423	28.29	5-Hydroxymethylfurfural	C_6_H_6_O_3_	126
20.024	22.17	1,2,3-Benzenetriol	C_6_H_6_O_3_	126
26.560	21.32	Cyclohexanol, 1R-4-trans-acetamido-2,3-trans-epoxy-	C_8_H_13_NO_3_	171
33.861	8.81	n-Hexadecanoic acid	C_16_H_32_O_2_	256
35.093	5.47	6-Methylheptanoic acid	C_8_H_16_O_2_	144

**Note:** The reported compounds represent the putative NIST library assignments. The cyclohexanol derivative should be considered a tentative assignment because structural confirmation using an authentic standard was not performed (Supplementary Material).

**Table 6:** Drug-like characteristics of GC–MS-putatively identified compounds from the MeOH leaf extract of *M. longifolia* were predicted using SwissADME.

Compound Name	PubChem CID	Formula	MW	Heavy Atoms	Rt Bonds	HBA	HBD	MR	TPSA	iLog P	ESOL Log S	ESOL Class
2,3-Dihydro-3,5-dihydroxy-6-methyl-4H-pyran-4-one	119838	C_6_H_8_O_4_	144.13	10	0	4	2	32.39	66.76	1.19	−0.5	VS
5-Hydroxymethylfurfural	237332	C_6_H_6_O_3_	126.11	9	2	3	1	30.22	50.44	0.91	−0.54	VS
1,2,3-Benzenetriol	1057	C_6_H_6_O_3_	126.11	9	0	3	3	32.51	60.69	0.97	−1.44	VS
Cyclohexanol, 1R-4-trans-acetamido-2,3-trans-epoxy-	537214	C_8_H_13_NO_3_	171.19	12	2	3	2	41.59	61.86	1.58	−0.29	VS
n-Hexadecanoic acid	985	C_16_H_32_O_2_	256.42	18	14	2	1	80.8	37.3	3.85	−5.02	MS
6-Methylheptanoic acid	90716	C_8_H_16_O_2_	144.21	10	5	2	1	42.34	37.3	2	−2.11	S

MW: Molecular weight, Rt bonds: Rotatable bonds, HBA: Hydrogen bond acceptor, HBD: Hydrogen bond doner, MR: Molar refractivity, TPSA: Topological surface area, VS: Very soluble, MS: Moderately soluble, S: Soluble.

**Table 7:** Pharmacokinetic and permeability profiles of GC-MS-putatively identified compounds from the MeOH leaf extract of *M. longifolia* were predicted using PreADMET.

Pub Chem CID	Aq. Solubility (mg/L)	BBB Permeant	Caco2 Cell Permeability (nm/sec)	MDCK Cell Permeability (nm/sec)	SP (logKp; cm/h)	PPB (%)	HIA (%)	Pgp Inhibitor	CYP-2D6 Inhibitor	CYP-2D6 Substrate
119838	8008.75	0.27	1.8	24.10	−4.76	38.43	75.91	Non	Non	Non
237332	43.39	0.52	6.42	152.57	−2.92	49.46	88.68	Non	Non	Non
1057	488,759	0.91	13.72	50.40	−3.50	100	77.41	Non	Non	Non
537214	74,860.5	0.27	16.61	1.04	−5.00	16.57	81.07	Non	Inhibitor	Non
985	136.24	8.21	26.07	70.51	−0.58	100	98.29	Inhibitor	Non	Non
90716	41,215.9	0.97	21.11	132.51	−1.65	100	93.71	Non	Non	Non

BBB: blood–brain barrier; SP: Skin permeability; PPB: plasma protein binding; HIA: human intestinal absorption.

**Table 8:** Toxicity assessment of the GC–MS-putatively identified compounds predicted using ProTox 3.0.

PubChem ID	LD_50_ (mg/kg)	Predicted Toxicity Class	Organ Toxicity					Toxicity End Point							
			Hepatotoxicity	Neurotoxicity	Nephrotoxicity	Respiratory Toxicity	Cardiotoxicity	Carcinogenicity	Immunotoxicity	Mutagenicity	Cytotoxicity	BBB-Barrier	Ecotoxicity	Clinical Toxicity	Nutritional Toxicity
119838	595	4	Inac	Inac	Act	Inac	Act	Inac	Inac	Act	Inac	Act	Inac	Act	Inac
237332	2500	5	Inac	Inac	Act	Act	Inac	Act	Inac	Act	Inac	Act	Inac	Inac	Inac
1057	300	3	Inac	Inac	Act	Inac	Act	Act	Inac	Act	Inac	Act	Inac	Inac	Inac
537214	5000	5	Inac	Inac	Act	Act	Inac	Inac	Inac	Inac	Inac	Act	Inac	Act	Inac
985	900	4	Inac	Inac	Inac	Inac	Inac	Inac	Inac	Inac	Inac	Act	Act	Inac	Inac
90716	900	4	Inac	Inac	Inac	Inac	Inac	Inac	Inac	Inac	Inac	Act	Act	Inac	Inac

Inac: Inactive (Predicted inactive); Act: Active (Predicted toxic) effect. **Note:** Green indicates a predicted inactive outcome, whereas red indicates a predicted active outcome. The predictions represent the model probabilities, not the experimental toxicity measurements. Darker and lighter shadings correspond to predicted probabilities of >70% and <70%, respectively.

**Table 9:** Molecular docking interaction profile of *M. longifolia* GC-MS-putatively identified compounds and the standard drug, acarbose, against α-amylase.

Compounds Name	Docking Score	Glide Score	Glide Emodel	Salt Bridge	H-Bond Involved	Amino Acid Involved
6-Methylheptanoic acid	−6.127	−6.132	−16.128	Arg^195^	Arg^195^; His^299^	Trp^58^, Trp^59^, Tyr^62^, Leu^162^, Leu^165^, Arg^195^, Asp^197^, Ala^198^, Glu^233^, His^299^, Asp^300^, and His^305^.
1,2,3-Benzenetriol	−4.748	−4.754	−24.132	---	Asp^197^; Glu^233^, Asp^300^	Trp^58^, Trp^59^, Tyr^62^, Val^98^, His^101^, Leu^162^, Leu^165^, Arg^195^, Asp^197^, Ala^198^, Glu^233^, Asp^300^, and His^305^.
Cyclohexanol, 1R-4-trans-acetamido-2,3-trans-epoxy-	−3.761	−3.761	−33.956	---	Asp^197^, Glu^233^	Trp^58^, Trp^59^, Tyr^62^, Gln^63^, Leu^162^, Leu^165^, Arg^195^, Asp^197^, Ala^198^, Glu^233^, His^299^, and Asp^300^.
2,3-Dihydro-3,5-dihydroxy-6-methyl-4H-pyran-4-one	−3.350	−4.449	−26.215	---	Asp^197^	Trp^58^, Trp^59^, Tyr^62^, Leu^162^, Leu^165^, Arg^195^, Asp^197^, Ala^198^, Glu^233^, His^299^, and Asp^300^.
5-Hydroxymethylfurfural	−2.843	−2.843	−25.066	---	His^299^	Trp^58^, Trp^59^, Tyr^62^, His^101^, Leu^165^, Arg^195^, Asp^197^, Glu^233^, His^299^, and Asp^300^.
n-Hexadecanoic acid	−2.205	−2.210	−27.631	Lys^200^	---	Trp^58^, Trp^59^, Tyr^62^, Glu^63^, His^101^, Gly^104^, Tyr^151^, Leu^162^, Thr^163^, Leu^165^, Asp^197^, Ala^198^, Lys^200^, His^201^, Glu^233^, Ile^235^, and Asp^300^.
* Acarbose	−5.77	−6.12	−72.250	---	Trp^59^, Asp^197^, Glu^240^, His^305^.	Trp^58^, Trp^59^, Tyr^62^, Gln^63^, His^101^, Tyr^151^, Leu^162^, Thr^163^, Leu^165^, Asp^197^, Ala^198^, Lys^200^, His^201^, Glu^233^, Ile^235^, Leu^237^, Glu^240^, Asp^300^, His^305^, Gly^306^, and Ala^307^.

* Standard drug.

## Data Availability

The data supporting the findings are provided in the manuscript and Supplementary Material, including the relevant GC–MS qualitative analysis data, original/native GC–MS files, and NIST library-search information. Intermediate or non-essential files were not provided separately. Additional scientifically relevant information, where available, can be obtained from the corresponding author upon reasonable request.

## Supplementary Materials

The following supporting information can be downloaded at https://www.mdpi.com/article/10.3390/plants15193059/s1, Figure S1: Superimposition of the native crystallographic and re-docked acarbose poses within the α-amylase active site (PDB ID: 3BAJ). The native crystallographic acarbose is shown in cyan, while the re-docked acarbose pose is shown in magenta. The close spatial overlap between the two poses illustrates the reproducibility of the docking protocol and was used for redocking validation; Table S1: SMILES of putatively identified compounds.
