# Supplementary material for: Phytochemical Profiling, In Vitro α-Amylase Inhibitory and Antioxidant Activities of Madhuca longifolia Leaf and Bark Extracts with Molecular Docking Analysis
Source: Plants (Basel). 2026 Oct 7;15(19):3059. doi: 10.3390/plants15193059 (PMC13666623; doi:10.3390/plants15193059)

Supplementary Materials

Phytochemical Profiling, In Vitro α-Amylase Inhibitory and Antioxidant Activities of Madhuca longifolia Leaf and Bark Extracts with Molecular Docking Analysis

1. Complete chromatogram of GC-MS

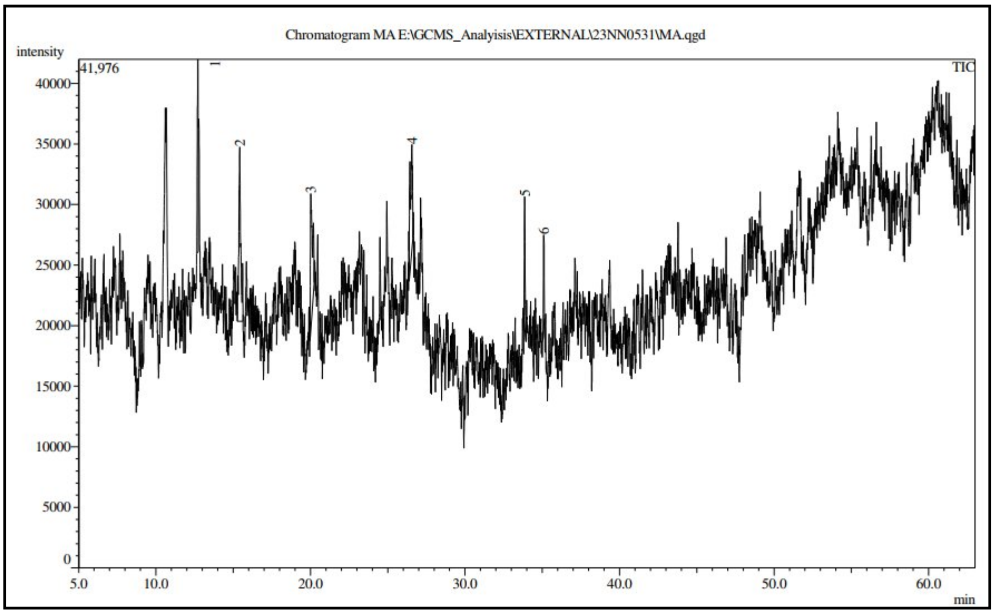

2. Peak Report TIC (Total Ion Chromatogram Peak Report)

Peak Report TIC

Peak#	R.Time	I.Time	F.Time	Area	Area%	Height	Height%	A/H	Mark	Name
1	12.715	12.642	12.742	60818	13.95	15889	23.09	3.83		4H-Pyran-4-one, 2,3-dihydro-3,5-dih
2	15.423	15.267	15.633	123365	28.29	14375	20.89	8.58	MI	5-Hydroxymethylfurfural
3	20.024	19.967	20.233	96693	22.17	11113	16.15	8.70		1,2,3-Benzenetriol
4	26.560	26.392	26.633	92964	21.32	8810	12.80	10.55	V	Cyclohexanol, 1R-4-trans-acetamido-
5	33.861	33.817	33.942	38401	8.81	10821	15.73	3.55		n-Hexadecanoic acid
6	35.093	35.050	35.142	23875	5.47	7802	11.34	3.06		6-Methylheptanoic acid
				436116	100.00	68810	100.00			

3. 2,3-Dihydro-3,5-dihydroxy-6-methyl-4H-pyran-4-one

Library

<< Target >>

Line#:1 R.Time:12.717(Scan#:927) MassPeaks:409

RawMode:Averaged 12.708-12.725(926-928) BasePeak:42.95(1710)

BG Mode:Calc. from Peak Group 1 - Event 1

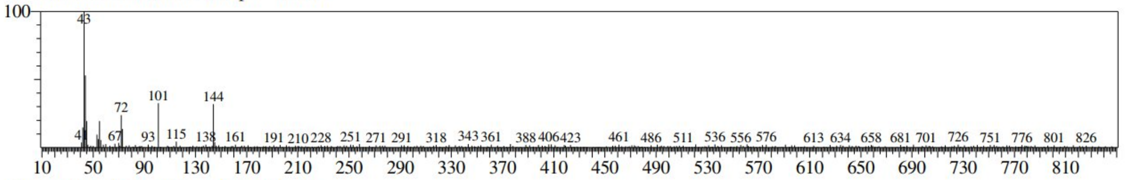

Hit#:1 Entry:14096 Library:NIST20M1.lib

SI:85 Formula:C6H8O4 CAS:28564-83-2 MolWeight:144 RetIndex:1269

CompName:4H-Pyran-4-one, 2,3-dihydro-3,5-dihydroxy-6-methyl- $$ 3,5-Dihydroxy-6-methyl-2,3-dihydro-4H-pyran-4-one $$ 2,3-dihydro-3,5-dihydroxy--

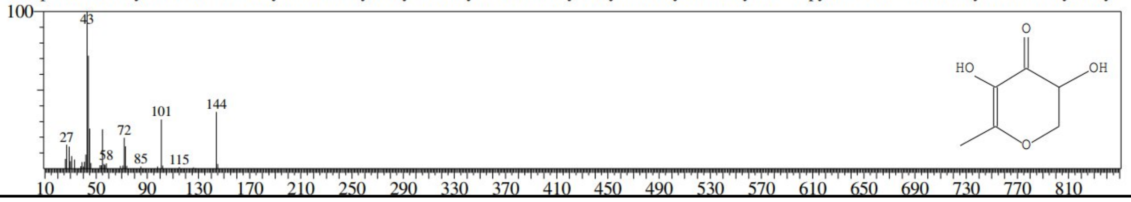

4. 5-Hydroxymethylfurfural

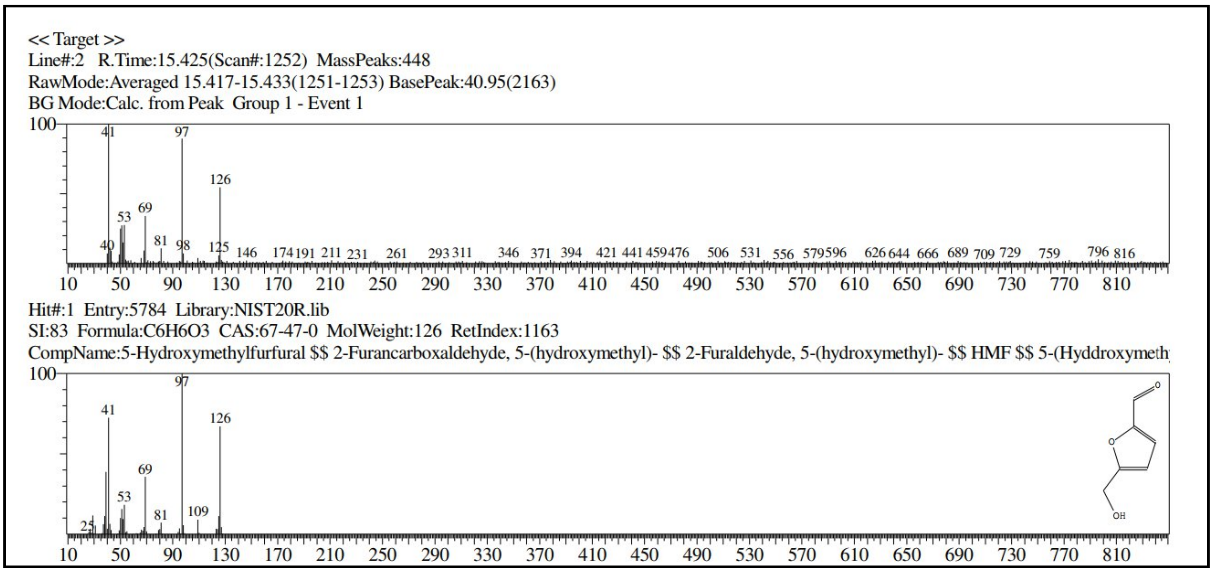

5. 1,2,3-Benzenetriol

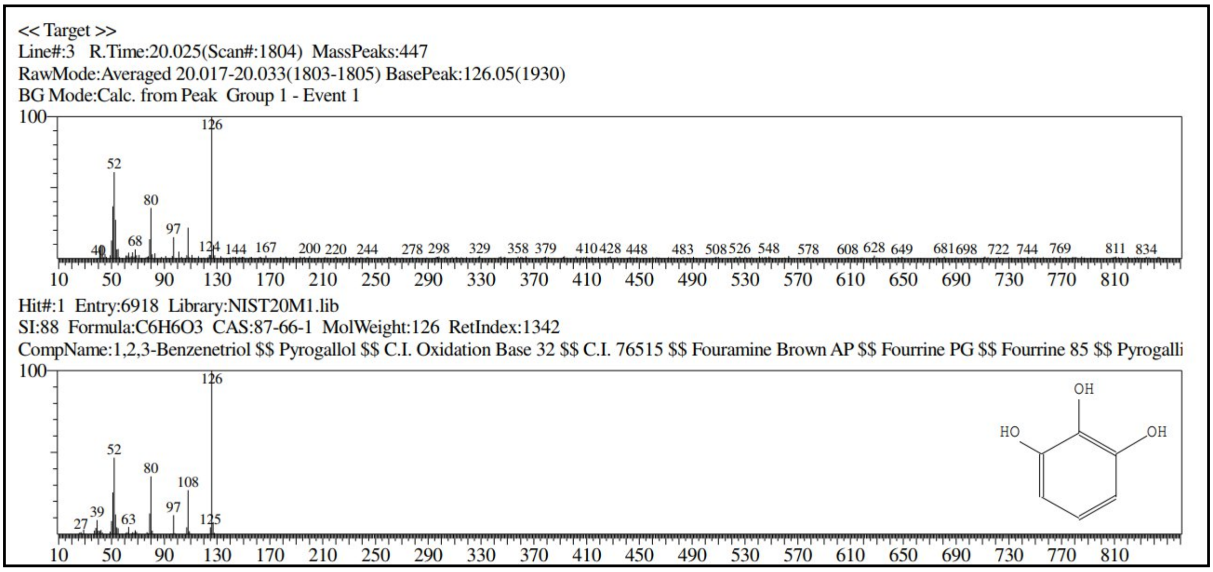

6. Cyclohexanol, 1R-4-trans-acetamido-2,3-trans-epoxy-

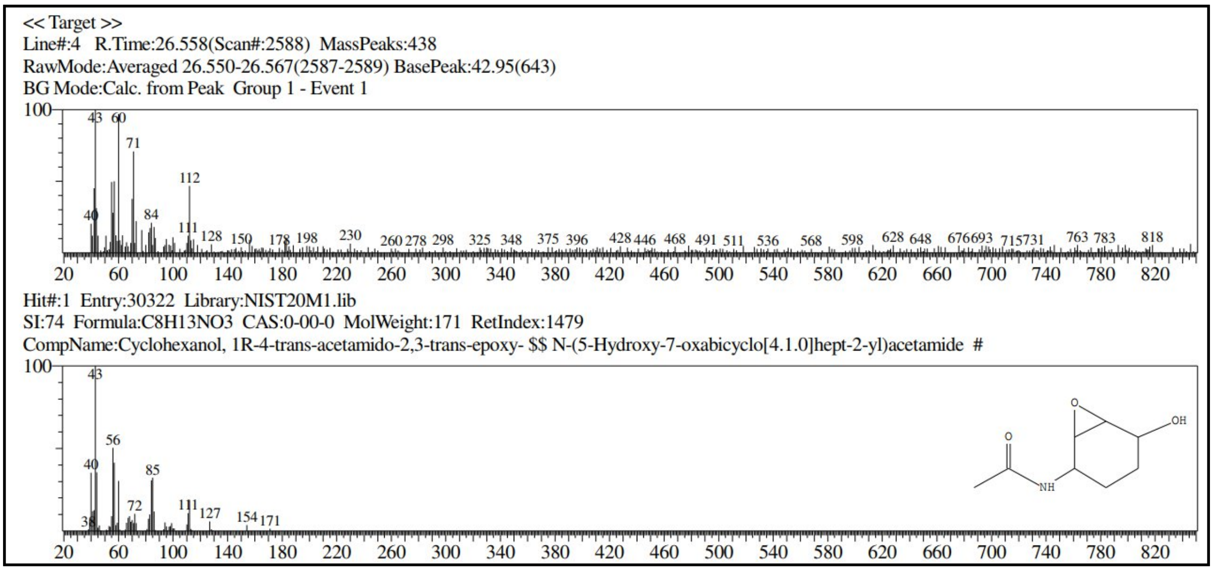

7. n-Hexadecanoic acid

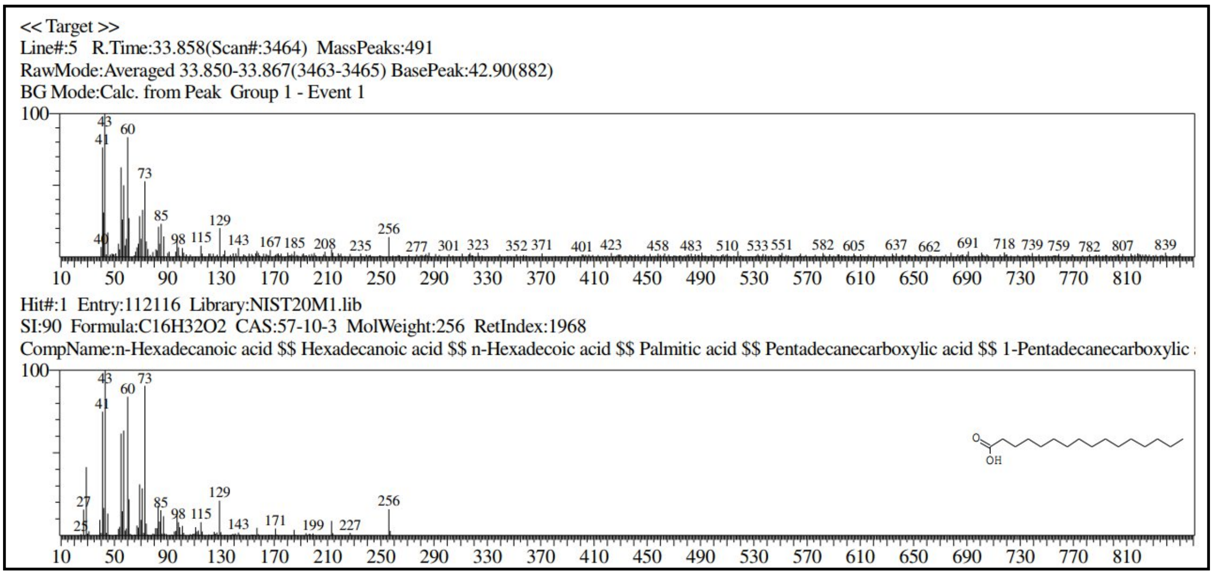

8. 6-Methylheptanoic acid

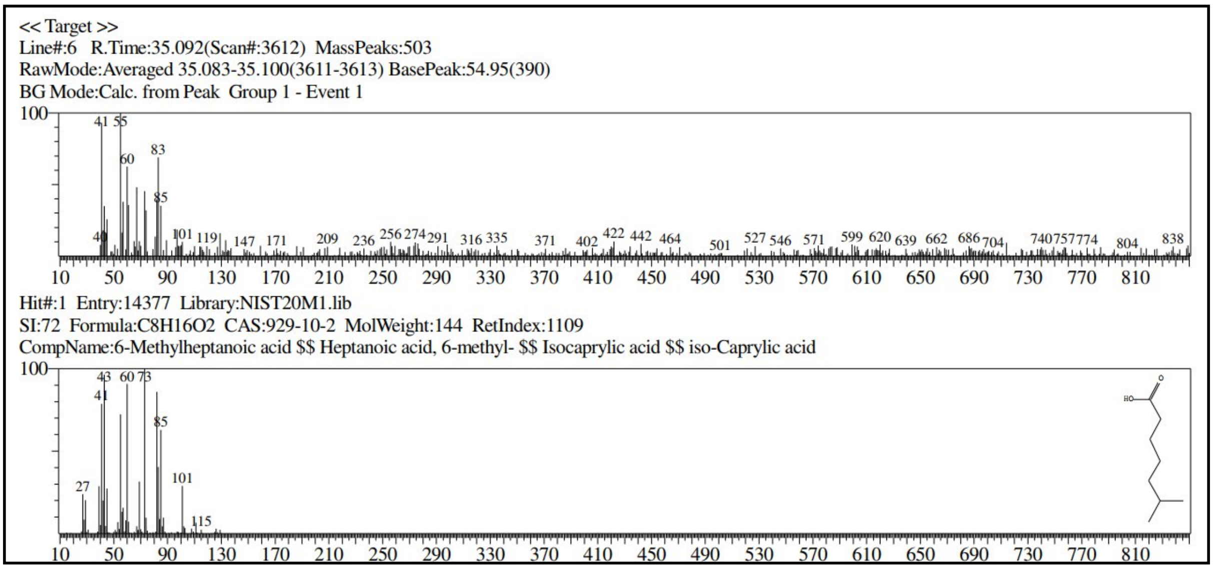

Table S1: SMILES of putatively identified compounds.

Compound name	CID	SMILES
2,3-Dihydro-3,5-dihydroxy-6-methyl-4H-pyran-4-one	119838	CC1=C(C(=O)C(CO1)O)O
5-Hydroxymethylfurfural	237332	C1=C(OC(=C1)C=O)CO
1,2,3-Benzenetriol	1057	C1=CC(=C(C(=C1)O)O)O
Cyclohexanol, 1R-4-trans-acetamido-2,3-trans-epoxy-	537214	CC(=O)NC1CCC(C2C1O2)O
n-Hexadecanoic acid	985	CCCCCCCCCCCCCCCC(=O)O
6-Methylheptanoic acid	90716	CC(C)CCCCC(=O)O

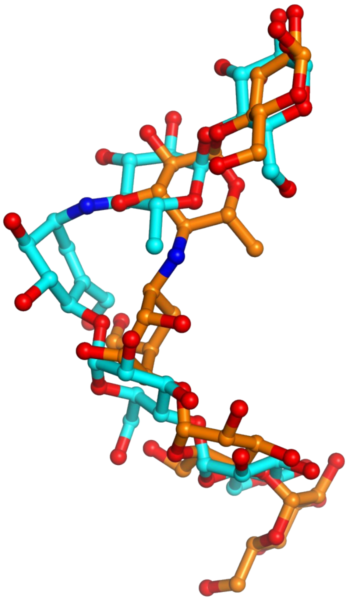

Figure S1: Superimposition of the native crystallographic and re-docked acarbose poses within the α-amylase active site (PDB ID: 3BAJ). The native crystallographic acarbose is shown in cyan, while the re-docked acarbose pose is shown in magenta. The close spatial overlap between the two poses illustrates the reproducibility of the docking protocol and was used for redocking validation.

Qualitative Analysis Report

Sample Information

Analyzed by	: Admin
Analyzed	: 6/10/2023 11:13:39 PM
Sample Type	: Unknown
Level #	: 1
Sample Name	: MA
Sample ID	:
IS Amount	: [1]=1
Sample Amount	: 1
Dilution Factor	: 1
Vial #	: 30
Injection Volume	: 1.00
Data File	: E:\GCMS_Analyisis\EXTERNAL\23NN0531\MA.qgd
Org Data File	: E:\GCMS_Analyisis\EXTERNAL\23NN0531\MA.qgd
Method File	: D:\gc-ms\MEthod\n-Alkanes_plant-extract.qgm
Org Method File	: D:\gc-ms\MEthod\n-Alkanes_plant-extract.qgm
Report File	:
Tuning File	: C:\GCMSsolution\System\Tune1\27052023_N_EI-F2_Column.qgt
Modified by	: Admin
Modified	: 6/12/2023 9:35:56 AM

Method

[Comment]

===== Analytical Line 1 =====

[AOC-20i+s]

# of Rinses with Presolvent	:5
# of Rinses with Solvent(post)	:5
# of Rinses with Sample	:3
Plunger Speed(Suction)	:High
Viscosity Comp. Time	:0.2 sec
Plunger Speed(Injection)	:High
Syringe Insertion Speed	:High
Injection Mode	:Normal
Pumping Times	:5
Inj. Port Dwell Time	:0.3 sec
Terminal Air Gap	:No
Plunger Washing Speed	:High
Washing Volume	:8uL
Syringe Suction Position	:0.0 mm
Syringe Injection Position	:0.0 mm
Solvent Selection	:All A,B,C

[GC-2010]

Column Oven Temp.	:70.0 °C
Injection Temp.	:280.00 °C
Injection Mode	:Splitless
Sampling Time	:1.00 min
Flow Control Mode	:Pressure
Pressure	:61.3 kPa
Total Flow	:14.0 mL/min
Column Flow	:1.00 mL/min
Linear Velocity	:36.7 cm/sec
Purge Flow	:3.0 mL/min
Split Ratio	:10.0
High Pressure Injection	:OFF
Carrier Gas Saver	:OFF

Oven Temp. Program

Rate	Temperature(°C)	Hold Time(min)
-	70.0	5.00
5.00	310.0	10.00

< Ready Check Heat Unit >

Column Oven	: Yes
SPL1	: Yes
MS	: Yes

< Ready Check Detector(FTD) >
< Ready Check Baseline Drift >
< Ready Check Injection Flow >

SPL1 Carrier	: Yes
SPL1 Purge	: Yes

< Ready Check APC Flow >
< Ready Check Detector APC Flow >

External Wait	:No
Equilibrium Time	:3.0 min

[GC Program]

[GCMS-QP2010 Ultra]

IonSourceTemp	:200.00 °C
Interface Temp.	:260.00 °C
Solvent Cut Time	:4.50 min
Detector Gain Mode	:Relative
Detector Gain	:1.26 kV +0.00 kV

Qualitative Analysis Report

Threshold :0

[MS Table]
--Group 1 - Event 1--
Start Time :5.00min
End Time :63.00min
ACQ Mode :Scan
Event Time :0.50sec
Scan Speed :1666
Start m/z :40.00
End m/z :850.00

Sample Inlet Unit :GC

[MS Program]
Use MS Program :OFF

Chromatogram MA E:\GCMS_Analyisis\EXTERNAL\23NN0531\MA.qgd

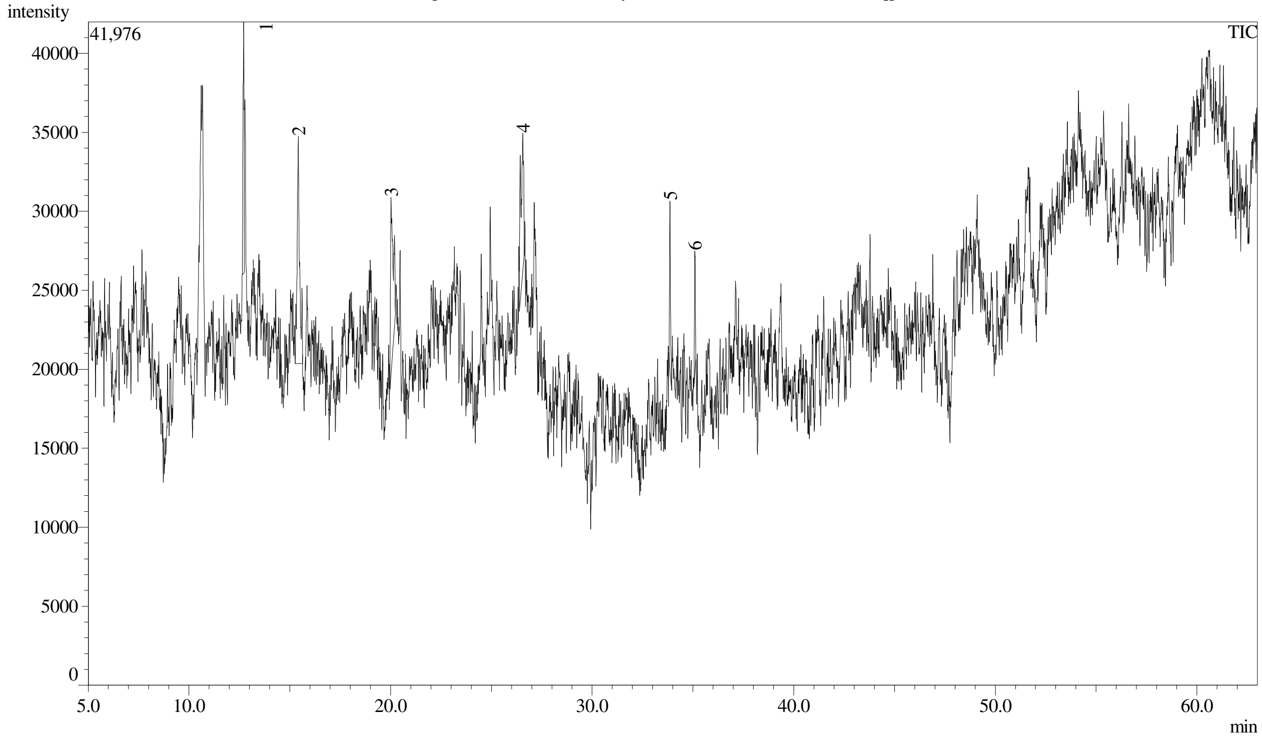

Peak Report TIC

Peak#	R.Time	I.Time	F.Time	Area	Area%	Height	Height%	A/H	Mark	Name
1	12.715	12.642	12.742	60818	13.95	15889	23.09	3.83		4H-Pyran-4-one, 2,3-dihydro-3,5-dih
2	15.423	15.267	15.633	123365	28.29	14375	20.89	8.58	MI	5-Hydroxymethylfurfural
3	20.024	19.967	20.233	96693	22.17	11113	16.15	8.70		1,2,3-Benzenetriol
4	26.560	26.392	26.633	92964	21.32	8810	12.80	10.55	V	Cyclohexanol, 1R-4-trans-acetamido-
5	33.861	33.817	33.942	38401	8.81	10821	15.73	3.55		n-Hexadecanoic acid
6	35.093	35.050	35.142	23875	5.47	7802	11.34	3.06		6-Methylheptanoic acid
				436116	100.00	68810	100.00			

Qualitative Analysis Report

Library

<< Target >>
Line#:1 R.Time:12.717(Scan#:927) MassPeaks:409
RawMode:Averaged 12.708-12.725(926-928) BasePeak:42.95(1710)
BG Mode:Calc. from Peak Group 1 - Event 1

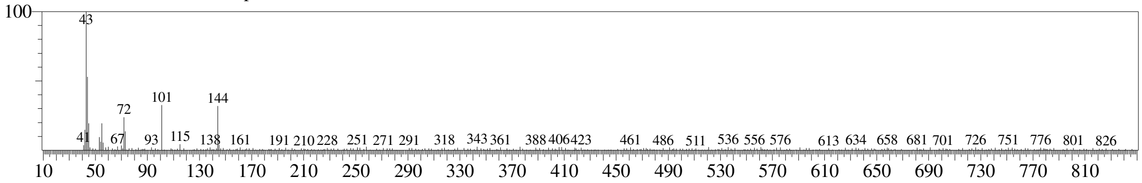

Hit#:1 Entry:14096 Library:NIST20M1.lib
SI:85 Formula:C6H8O4 CAS:28564-83-2 MolWeight:144 RetIndex:1269
CompName:4H-Pyran-4-one, 2,3-dihydro-3,5-dihydroxy-6-methyl- $$ 3,5-Dihydroxy-6-methyl-2,3-dihydro-4H-pyran-4-one $$ 2,3-dihydro-3,5-dihydroxy-

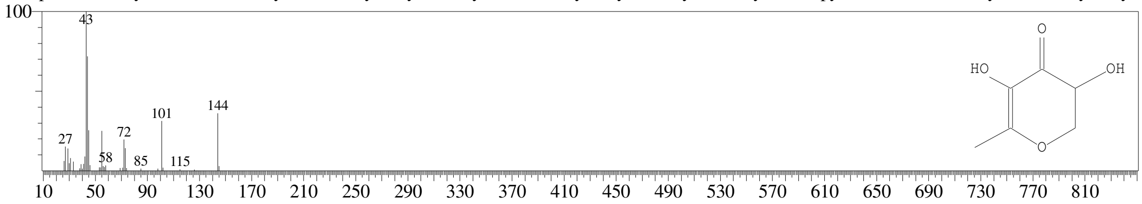

Hit#:2 Entry:9624 Library:NIST20R.lib
SI:84 Formula:C6H8O4 CAS:28564-83-2 MolWeight:144 RetIndex:1269
CompName:4H-Pyran-4-one, 2,3-dihydro-3,5-dihydroxy-6-methyl- $$ 3,5-Dihydroxy-6-methyl-2,3-dihydro-4H-pyran-4-one $$ 2,3-dihydro-3,5-dihydroxy-

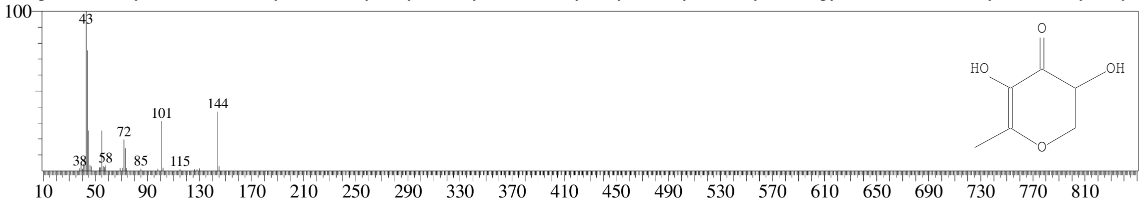

Hit#:3 Entry:9625 Library:NIST20R.lib
SI:83 Formula:C6H8O4 CAS:28564-83-2 MolWeight:144 RetIndex:1269
CompName:4H-Pyran-4-one, 2,3-dihydro-3,5-dihydroxy-6-methyl- $$ 3,5-Dihydroxy-6-methyl-2,3-dihydro-4H-pyran-4-one $$ 2,3-dihydro-3,5-dihydroxy-

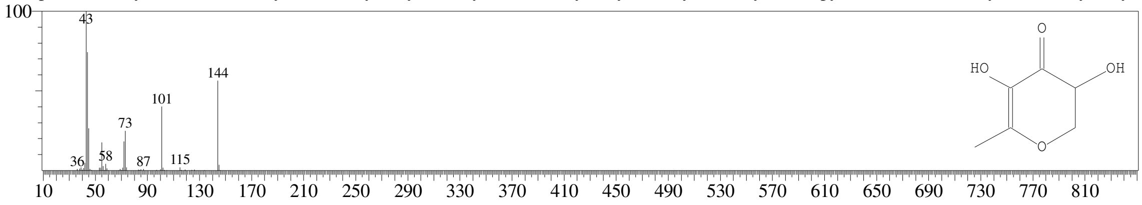

Hit#:4 Entry:9626 Library:NIST20R.lib
SI:80 Formula:C6H8O4 CAS:28564-83-2 MolWeight:144 RetIndex:1269
CompName:4H-Pyran-4-one, 2,3-dihydro-3,5-dihydroxy-6-methyl- $$ 3,5-Dihydroxy-6-methyl-2,3-dihydro-4H-pyran-4-one $$ 2,3-dihydro-3,5-dihydroxy-

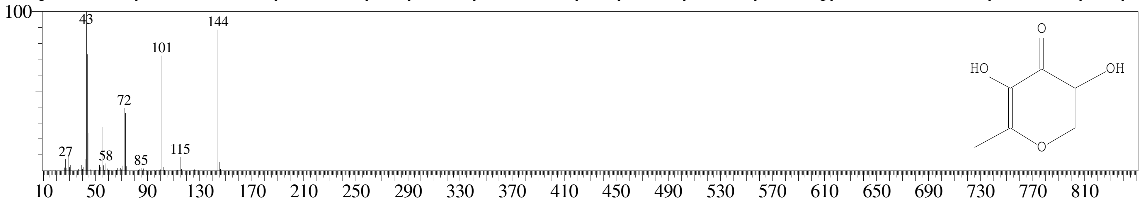

Hit#:5 Entry:4877 Library:NIST20M1.lib
SI:74 Formula:C6H12O2 CAS:3299-32-9 MolWeight:116 RetIndex:761
CompName:1,3-Dioxolane, 2,4,5-trimethyl- $$ 2,4,5-Trimethyl-1,3-dioxolane #

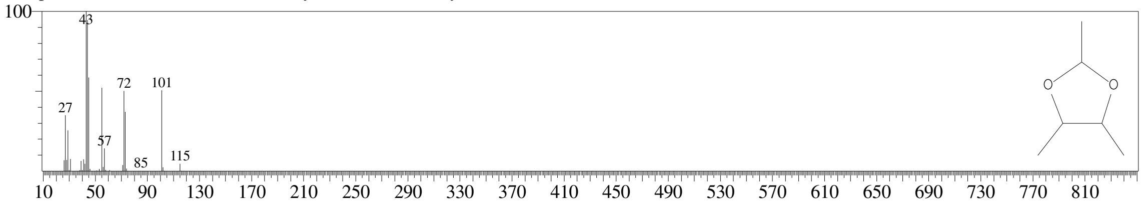

Qualitative Analysis Report

<< Target >>
Line#:2 R.Time:15.425(Scan#:1252) MassPeaks:448
RawMode:Averaged 15.417-15.433(1251-1253) BasePeak:40.95(2163)
BG Mode:Calc. from Peak Group 1 - Event 1

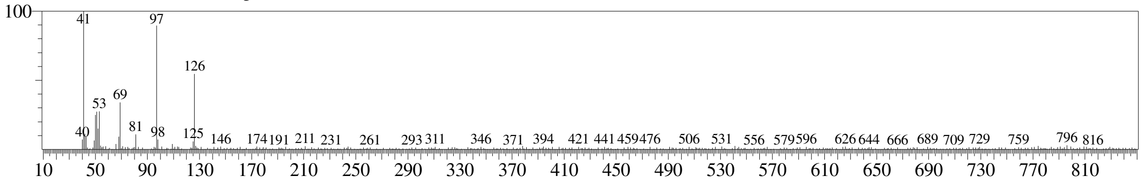

Hit#:1 Entry:5784 Library:NIST20R.lib
SI:83 Formula:C6H6O3 CAS:67-47-0 MolWeight:126 RetIndex:1163
CompName:5-Hydroxymethylfurfural $$ 2-Furancarboxaldehyde, 5-(hydroxymethyl)- $$ 2-Furaldehyde, 5-(hydroxymethyl)- $$ HMF $$ 5-(Hyddroxymeth

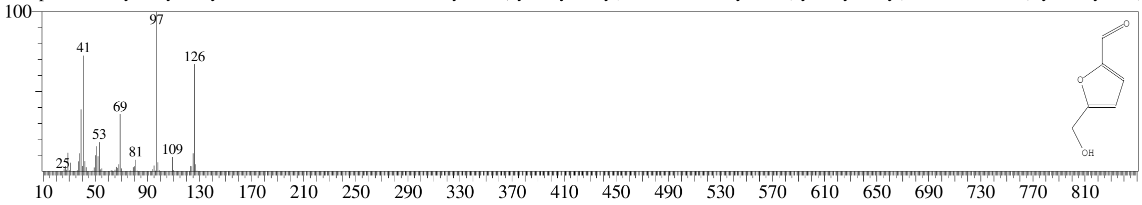

Hit#:2 Entry:6911 Library:NIST20M1.lib
SI:83 Formula:C6H6O3 CAS:67-47-0 MolWeight:126 RetIndex:1163
CompName:5-Hydroxymethylfurfural $$ 2-Furancarboxaldehyde, 5-(hydroxymethyl)- $$ 2-Furaldehyde, 5-(hydroxymethyl)- $$ HMF $$ 5-(Hyddroxymeth

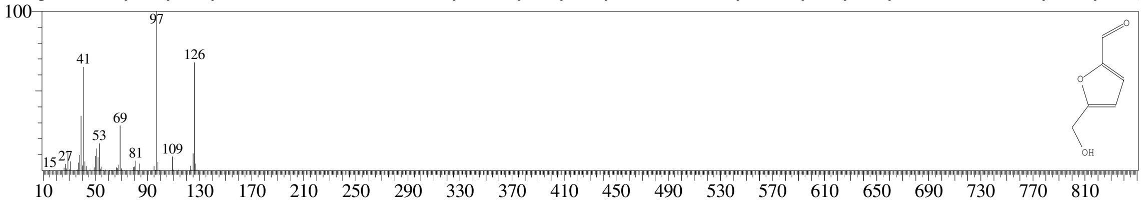

Hit#:3 Entry:5783 Library:NIST20R.lib
SI:81 Formula:C6H6O3 CAS:67-47-0 MolWeight:126 RetIndex:1163
CompName:5-Hydroxymethylfurfural $$ 2-Furancarboxaldehyde, 5-(hydroxymethyl)- $$ 2-Furaldehyde, 5-(hydroxymethyl)- $$ HMF $$ 5-(Hyddroxymeth

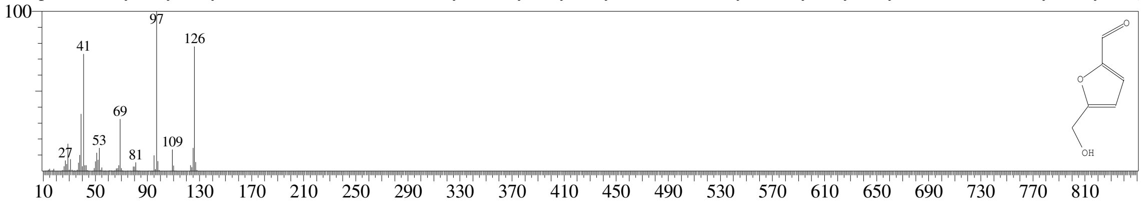

Hit#:4 Entry:5785 Library:NIST20R.lib
SI:81 Formula:C6H6O3 CAS:67-47-0 MolWeight:126 RetIndex:1163
CompName:5-Hydroxymethylfurfural $$ 2-Furancarboxaldehyde, 5-(hydroxymethyl)- $$ 2-Furaldehyde, 5-(hydroxymethyl)- $$ HMF $$ 5-(Hyddroxymeth

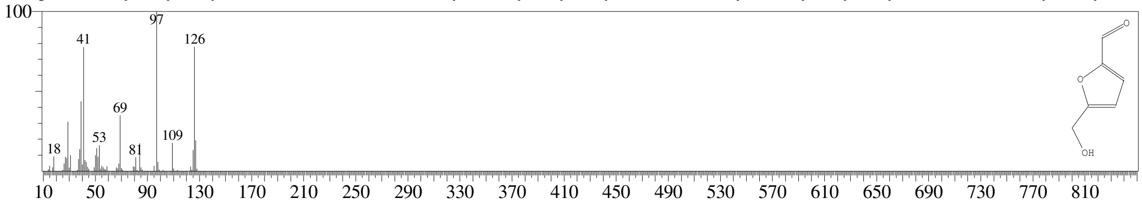

Hit#:5 Entry:5782 Library:NIST20R.lib
SI:80 Formula:C6H6O3 CAS:67-47-0 MolWeight:126 RetIndex:1163
CompName:5-Hydroxymethylfurfural $$ 2-Furancarboxaldehyde, 5-(hydroxymethyl)- $$ 2-Furaldehyde, 5-(hydroxymethyl)- $$ HMF $$ 5-(Hyddroxymeth

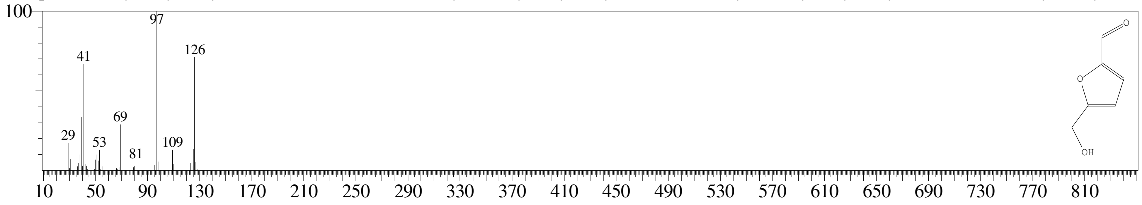

Qualitative Analysis Report

<< Target >>
Line#:3 R.Time:20.025(Scan#:1804) MassPeaks:447
RawMode:Averaged 20.017-20.033(1803-1805) BasePeak:126.05(1930)
BG Mode:Calc. from Peak Group 1 - Event 1

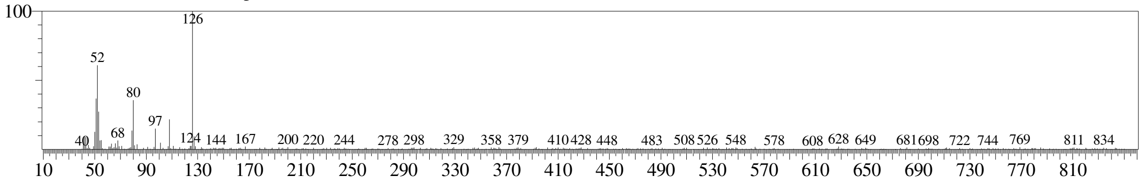

Hit#:1 Entry:6918 Library:NIST20M1.lib
SI:88 Formula:C6H6O3 CAS:87-66-1 MolWeight:126 RetIndex:1342
CompName:1,2,3-Benzenetriol $$ Pyrogallol $$ C.I. Oxidation Base 32 $$ C.I. 76515 $$ Fouramine Brown AP $$ Fourrine PG $$ Fourrine 85 $$ Pyrogalli

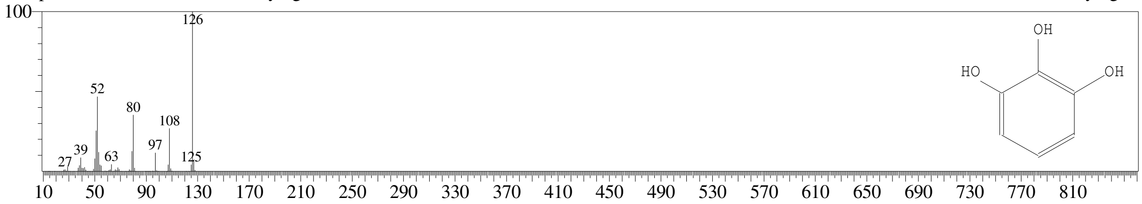

Hit#:2 Entry:5794 Library:NIST20R.lib
SI:86 Formula:C6H6O3 CAS:87-66-1 MolWeight:126 RetIndex:1342
CompName:1,2,3-Benzenetriol $$ Pyrogallol $$ C.I. Oxidation Base 32 $$ C.I. 76515 $$ Fouramine Brown AP $$ Fourrine PG $$ Fourrine 85 $$ Pyrogalli

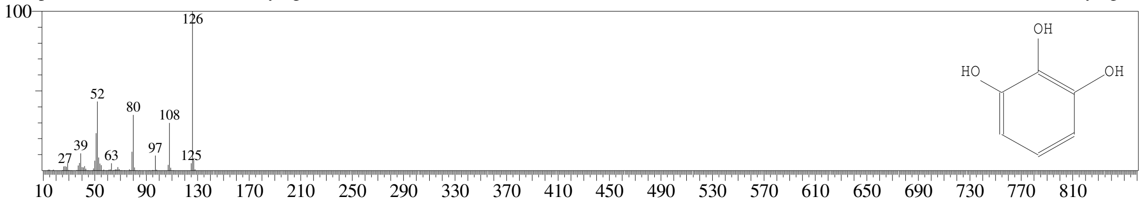

Hit#:3 Entry:5793 Library:NIST20R.lib
SI:85 Formula:C6H6O3 CAS:87-66-1 MolWeight:126 RetIndex:1342
CompName:1,2,3-Benzenetriol $$ Pyrogallol $$ C.I. Oxidation Base 32 $$ C.I. 76515 $$ Fouramine Brown AP $$ Fourrine PG $$ Fourrine 85 $$ Pyrogalli

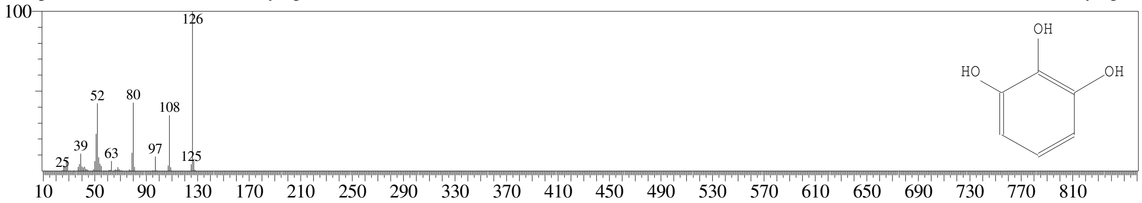

Hit#:4 Entry:5773 Library:NIST20R.lib
SI:84 Formula:C6H6O3 CAS:533-73-3 MolWeight:126 RetIndex:1342
CompName:1,2,4-Benzenetriol $$ 1,3,4-Benzenetriol $$ 2,5-Dihydroxyphenol $$ Hydroxyhydroquinone $$ 2-Hydroxy-1,4-hydroquinone $$ 1,2,4-Trihydro

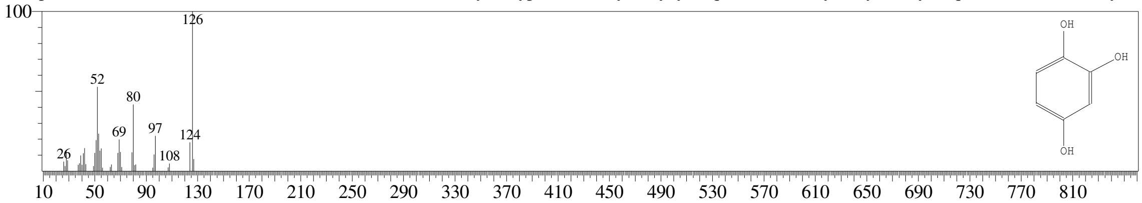

Hit#:5 Entry:6903 Library:NIST20M1.lib
SI:83 Formula:C6H6O3 CAS:533-73-3 MolWeight:126 RetIndex:1342
CompName:1,2,4-Benzenetriol $$ 1,3,4-Benzenetriol $$ 2,5-Dihydroxyphenol $$ Hydroxyhydroquinone $$ 2-Hydroxy-1,4-hydroquinone $$ 1,2,4-Trihydro

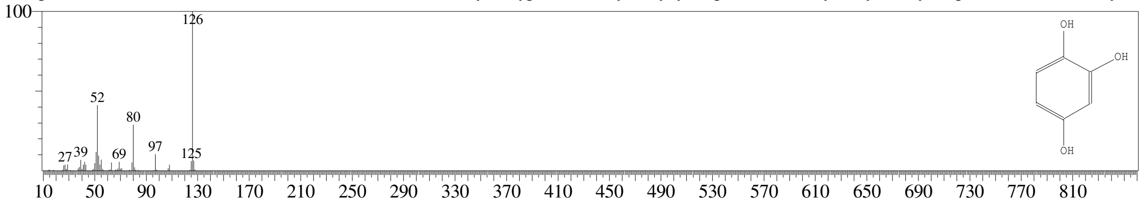

Qualitative Analysis Report

<< Target >>
Line#:4 R.Time:26.558(Scan#:2588) MassPeaks:438
RawMode:Averaged 26.550-26.567(2587-2589) BasePeak:42.95(643)
BG Mode:Calc. from Peak Group 1 - Event 1

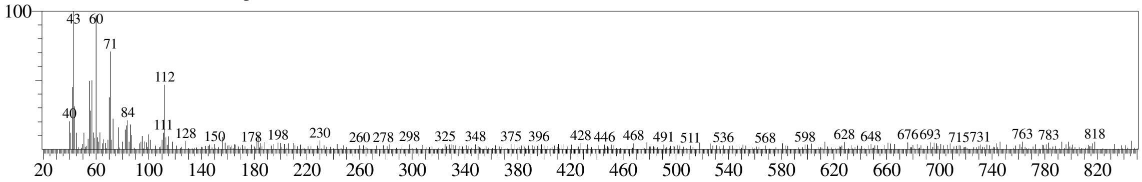

Hit#:1 Entry:30322 Library:NIST20M1.lib
SI:74 Formula:C8H13NO3 CAS:0-00-0 MolWeight:171 RetIndex:1479
CompName:Cyclohexanol, 1R-4-trans-acetamido-2,3-trans-epoxy- $$ N-(5-Hydroxy-7-oxabicyclo[4.1.0]hept-2-yl)acetamide #

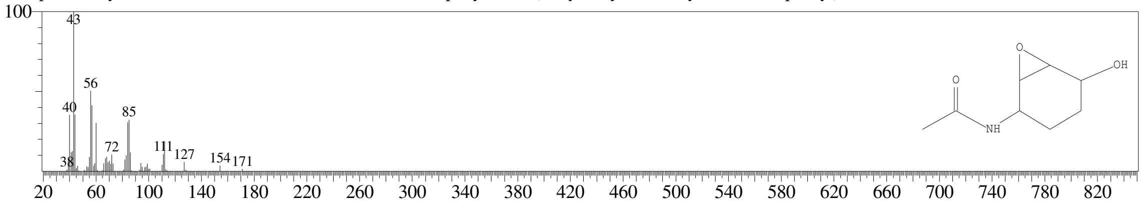

Hit#:2 Entry:68573 Library:NIST20M1.lib
SI:73 Formula:C12H24O3 CAS:1883-13-2 MolWeight:216 RetIndex:1733
CompName:Dodecanoic acid, 3-hydroxy- $$ .beta.-Hydroxydodecanoic acid $$ .beta.-Hydroxylauric acid $$ 3-Hydroxydodecanoic acid $$ 3-Hydroxylauri

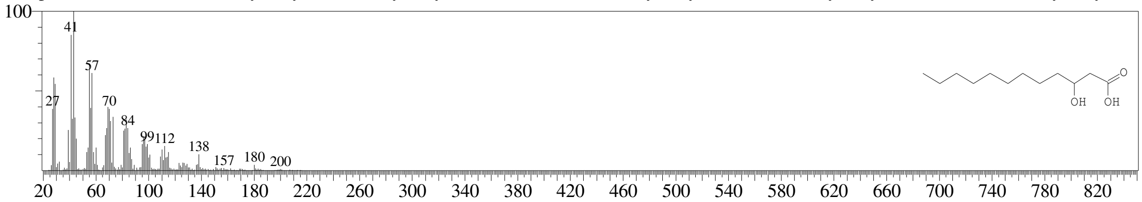

Hit#:3 Entry:144795 Library:NIST20M1.lib
SI:73 Formula:C18H36O2 CAS:73947-30-5 MolWeight:284 RetIndex:1914
CompName:Decanoic acid, 2-ethylhexyl ester $$ 2-Ethylhexyl decanoate #

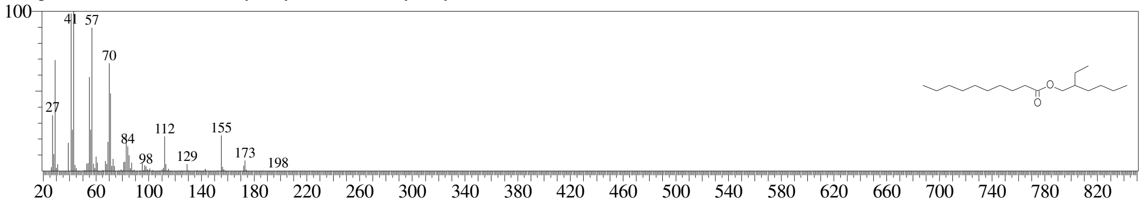

Hit#:4 Entry:14544 Library:NIST20M1.lib
SI:72 Formula:C9H20O CAS:98982-97-9 MolWeight:144 RetIndex:1030
CompName:1-Heptanol, 2,4-dimethyl-,

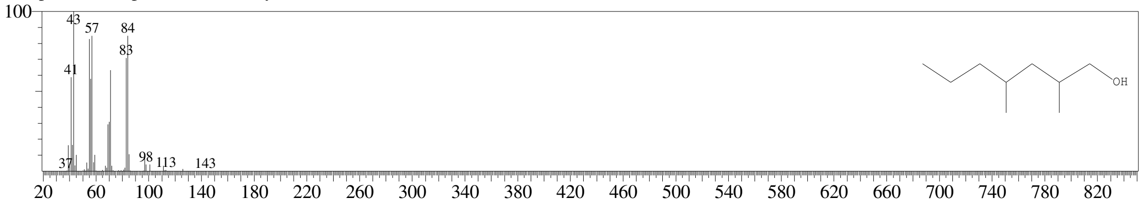

Hit#:5 Entry:30321 Library:NIST20M1.lib
SI:72 Formula:C8H13NO3 CAS:77803-84-0 MolWeight:171 RetIndex:1479
CompName:Cyclohexanol, 1R-4-acetamido-2,3-cis-epoxy- $$ N-(5-Hydroxy-7-oxabicyclo[4.1.0]hept-2-yl)acetamide #

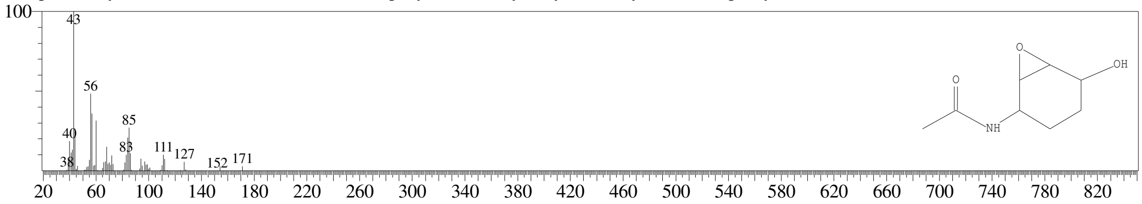

Qualitative Analysis Report

<< Target >>
Line#:5 R.Time:33.858(Scan#:3464) MassPeaks:491
RawMode:Averaged 33.850-33.867(3463-3465) BasePeak:42.90(882)
BG Mode:Calc. from Peak Group 1 - Event 1

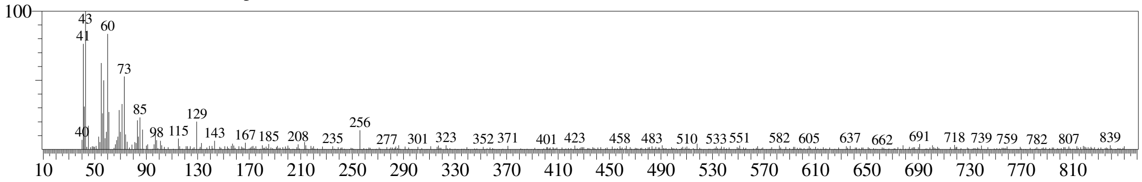

Hit#:1 Entry:112116 Library:NIST20M1.lib
SI:90 Formula:C16H32O2 CAS:57-10-3 MolWeight:256 RetIndex:1968
CompName:n-Hexadecanoic acid $$ Hexadecanoic acid $$ n-Hexadecoic acid $$ Palmitic acid $$ Pentadecanecarboxylic acid $$ 1-Pentadecanecarboxylic

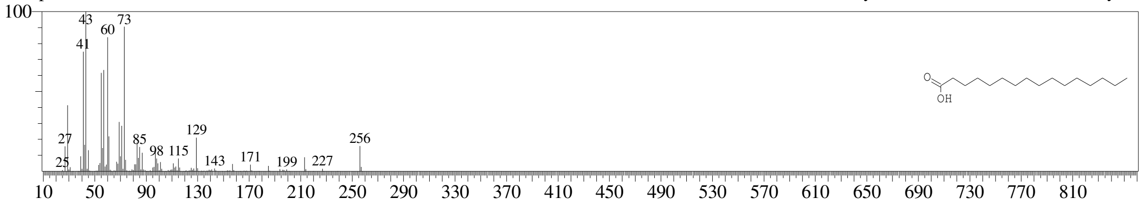

Hit#:2 Entry:31598 Library:NIST20R.lib
SI:88 Formula:C16H32O2 CAS:57-10-3 MolWeight:256 RetIndex:1968
CompName:n-Hexadecanoic acid $$ Hexadecanoic acid $$ n-Hexadecoic acid $$ Palmitic acid $$ Pentadecanecarboxylic acid $$ 1-Pentadecanecarboxylic

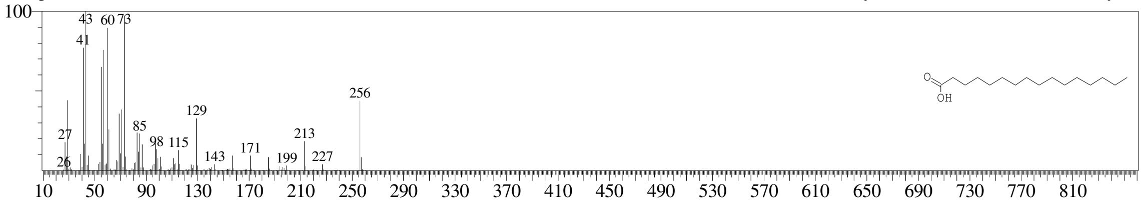

Hit#:3 Entry:29888 Library:NIST20R.lib
SI:87 Formula:C15H30O2 CAS:1002-84-2 MolWeight:242 RetIndex:1869
CompName:Pentadecanoic acid $$ Pentadecylic acid $$ n-Pentadecanoic acid $$ n-Pentadecylic acid

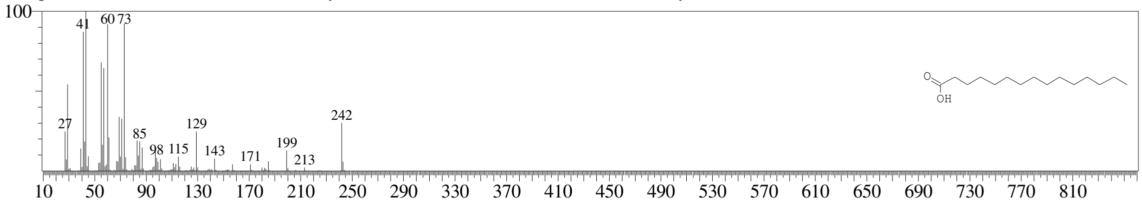

Hit#:4 Entry:19776 Library:NIST20R.lib
SI:87 Formula:C11H22O2 CAS:112-37-8 MolWeight:186 RetIndex:1471
CompName:Undecanoic acid $$ n-Undecanoic acid $$ n-Undecoic acid $$ n-Undecylic acid $$ Hendecanoic acid $$ Undecylic acid $$ 1-Decanecarboxylic

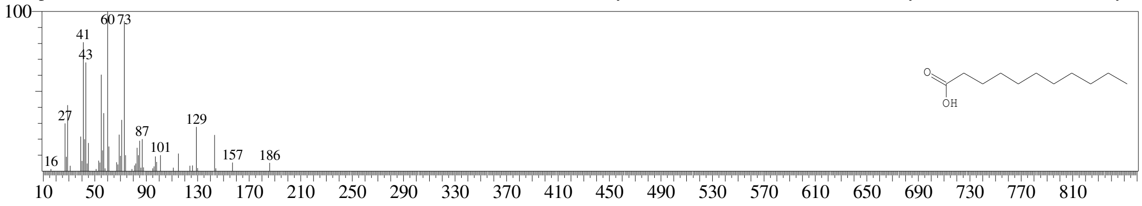

Hit#:5 Entry:34461 Library:NIST20R.lib
SI:86 Formula:C18H36O2 CAS:57-11-4 MolWeight:284 RetIndex:2167
CompName:Octadecanoic acid $$ Stearic acid $$ n-Octadecanoic acid $$ Humko Industrene R $$ Hydrofol Acid 150 $$ Hystrene S-97 $$ Hystrene T-70 $

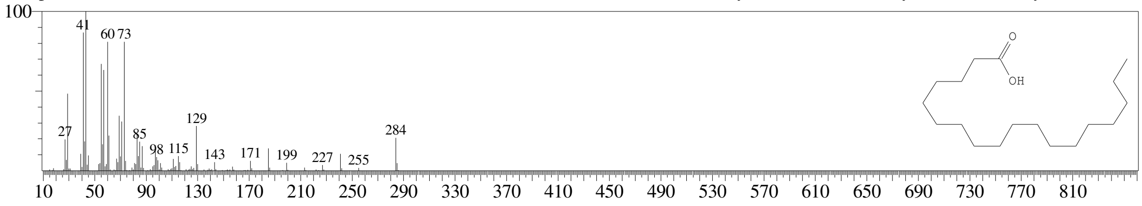

Qualitative Analysis Report

<< Target >>
Line#:6 R.Time:35.092(Scan#:3612) MassPeaks:503
RawMode:Averaged 35.083-35.100(3611-3613) BasePeak:54.95(390)
BG Mode:Calc. from Peak Group 1 - Event 1

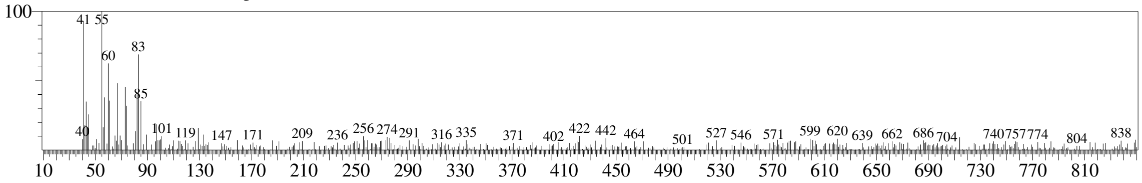

Hit#:1 Entry:14377 Library:NIST20M1.lib
SI:72 Formula:C8H16O2 CAS:929-10-2 MolWeight:144 RetIndex:1109
CompName:6-Methylheptanoic acid $$ Heptanoic acid, 6-methyl- $$ Isocaprylic acid $$ iso-Caprylic acid

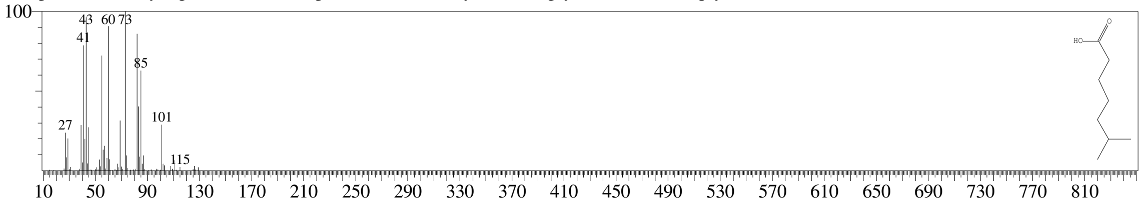

Hit#:2 Entry:31092 Library:NIST20M1.lib
SI:70 Formula:C9H16O3 CAS:2553-17-5 MolWeight:172 RetIndex:1461
CompName:9-Oxononanoic acid

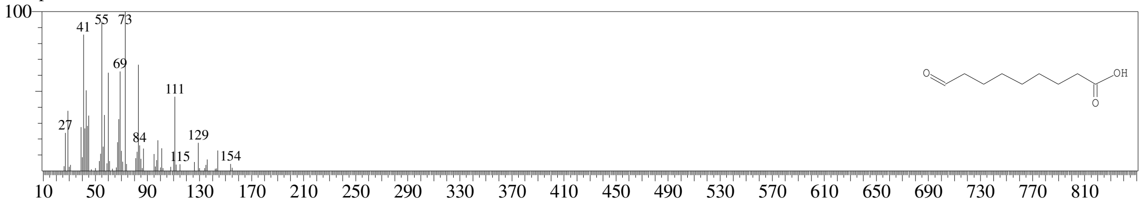

Hit#:3 Entry:21938 Library:NIST20M1.lib
SI:70 Formula:C9H18O2 CAS:54947-74-9 MolWeight:158 RetIndex:1208
CompName:4-Methyloctanoic acid $$ Octanoic acid, 4-methyl-

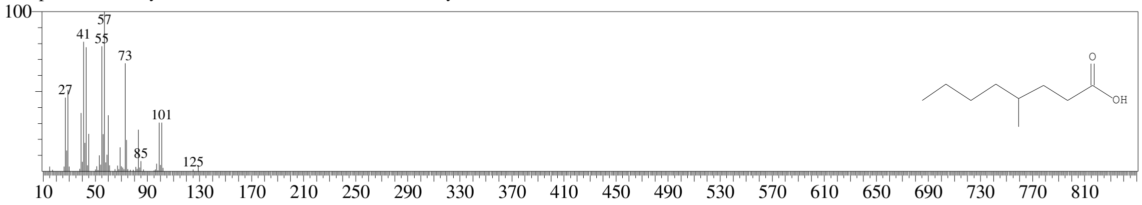

Hit#:4 Entry:35751 Library:NIST20R.lib
SI:69 Formula:C19H38O2 CAS:646-30-0 MolWeight:298 RetIndex:2266
CompName:Nonadecanoic acid $$ n-Nonadecanoic acid

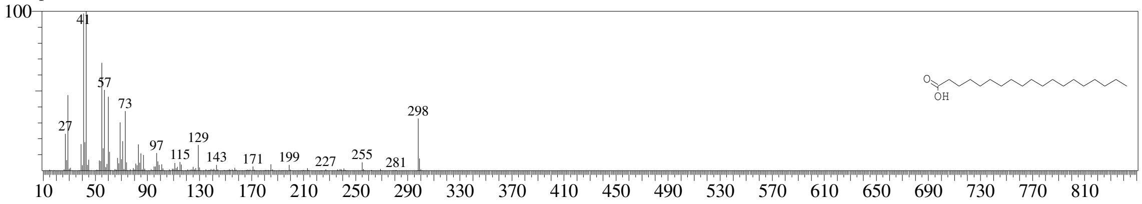

Hit#:5 Entry:19776 Library:NIST20R.lib
SI:69 Formula:C11H22O2 CAS:112-37-8 MolWeight:186 RetIndex:1471
CompName:Undecanoic acid $$ n-Undecanoic acid $$ n-Undecoic acid $$ n-Undecylic acid $$ Hendecanoic acid $$ Undecylic acid $$ 1-Decanecarboxylic

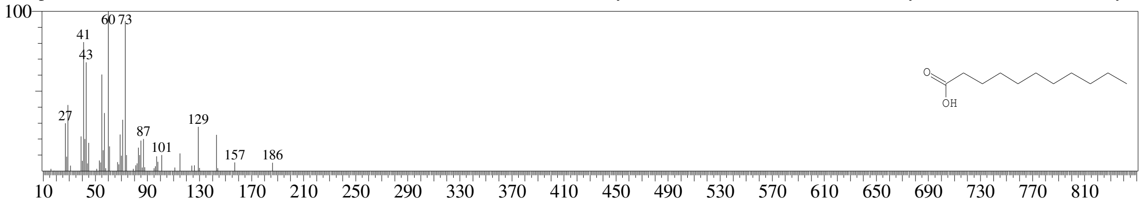

Supplement: Supplementary file 1 [file plants-15-03059-s001.zip › plants-4526456-supplementary.pdf]
